# Shared and language-specific phonological processing in the human temporal lobe

**DOI:** 10.1038/s41586-025-09748-8

**Published:** 2025-11-19

**Authors:** Ilina Bhaya-Grossman, Matthew K. Leonard, Yizhen Zhang, Laura Gwilliams, Keith Johnson, Junfeng Lu, Edward F. Chang

**Affiliations:** 1 Department of Neurological Surgery, University of California, San Francisco, CA USA; 2 Weill Institute for Neuroscience, University of California, San Francisco, CA USA; 3 Joint Program in Bioengineering, University of California, Berkeley and University of California, San Francisco, Berkeley, CA USA; 4 Department of Linguistics, University of California, Berkeley, Berkeley, CA USA; 5 Department of Neurosurgery, Huashan Hospital, Fudan University, Shanghai, China

**Keywords:** Language, Neural encoding, Perception

## Abstract

All spoken languages are produced by the human vocal tract, which defines the limited set of possible speech sounds. Despite this constraint, however, there exists incredible diversity in the world’s 7,000 spoken languages, each of which is learned through extensive experience hearing speech in language-specific contexts^1^. It remains unknown which elements of speech processing in the brain depend on daily language experience and which do not. In this study, we recorded high-density cortical activity from adult participants with diverse language backgrounds as they listened to speech in their native language and an unfamiliar foreign language. We found that, regardless of language experience, both native and foreign languages elicited similar cortical responses in the superior temporal gyrus (STG), associated with shared acoustic–phonetic processing of foundational speech sound features^2,3^, such as vowels and consonants. However, only during native language listening did we observe enhanced neural encoding in the STG for word boundaries, word frequency and language-specific sound sequence statistics. In a separate cohort of bilingual participants, this encoding of word- and sequence-level information appeared for both familiar languages in the same individual and in the same STG neural populations. These results indicate that experience-dependent language processing involves dynamic integration of both shared acoustic–phonetic and language-specific sequence- and word-level information in the STG.

## Abstract (web summary)

The human superior temporal gyrus processes acoustic–phonetic properties of speech regardless of whether the language is familiar to the listener, but only encodes word boundaries and language-specific sound sequences if the language is known.

## Main

When listening to speech in a foreign language, we hear a fast, continuous and uninterpretable stream of speech sounds^4,5^. However, when we listen to speech in a language we know, we hear these sounds as words. This specialized perception of speech in one’s native languages develops over the course of language acquisition^4,6–11^. A central question in speech neuroscience is how and to what extent the adult brain’s processing of speech is specific to one’s native languages.

Previous work has identified the human STG, a non-primary auditory area, as crucial for speech perception^3,12–14^. Both native and foreign or unfamiliar speech elicit a broadly similar pattern of activation in the bilateral middle STG^15–23^. As a result, models of speech processing have hypothesized that the middle STG performs similar complex acoustic processing across native and foreign speech^13,24^. However, it is unclear to what extent acoustic–phonetic and higher-level speech representations in STG are affected by language experience, particularly given that language-specific features such as phonetic categories^2,25,26^ and lexical tone^27,28^ are encoded there. Furthermore, STG neural populations have been shown to encode aspects of whole words^3,29^, which are highly specific to individual languages and must be learned through extensive exposure. In this study, we ask which features are affected by language experience in the human temporal cortex during natural speech processing.

**Fig. 1:**
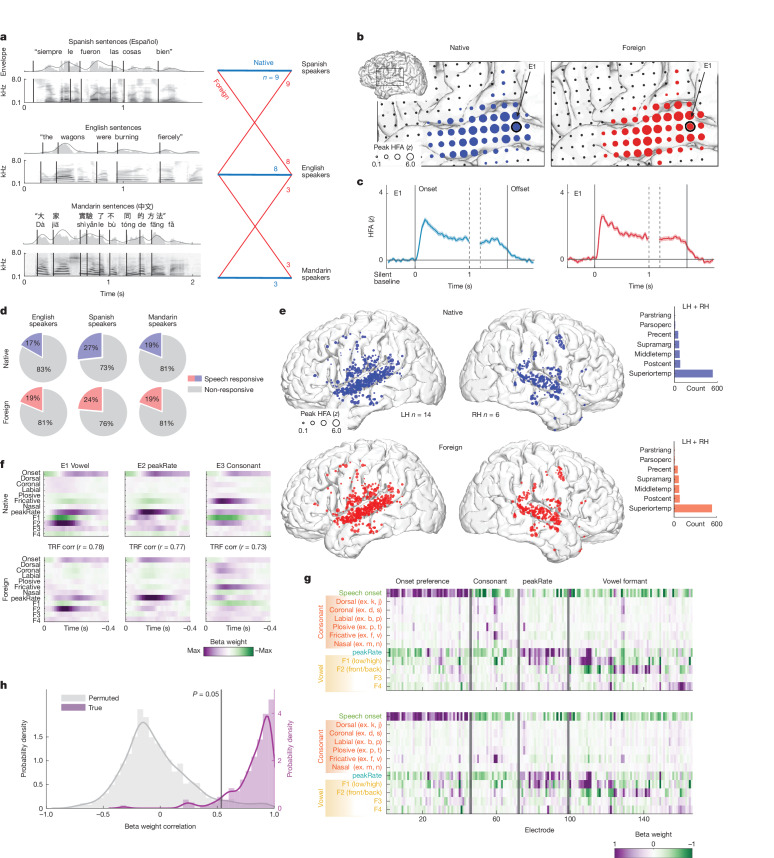
Shared acoustic–phonetic processing in STG across native and foreign speech. **a**, Spanish-, English- and Mandarin-speaking ECoG participants passively listened to native and foreign speech. Left, sound envelope and spectrogram of example stimuli. Right, coloured lines indicate speech condition, labelled with the number of participants in each group. **b**, In an example Spanish listener, the same neural populations are active in response to native (left) and foreign (right) speech. Electrode size indicates magnitude of the peak HFA averaged sentence response; colour indicates speech condition. **c**, Average HFA sentence response for an example electrode is highly correlated across native and foreign speech (Pearson *r*(250) = 0.986, *P* < 0.001). Neural responses time-aligned to sentence onset and offset; colour indicates speech condition. Shaded patches indicate standard error of the mean across sentences. **d**, Proportions of speech-responsive electrodes for Spanish, English and Mandarin speakers are similar across native (top) and foreign (bottom) speech, ranging from 17% to 27% across groups. **e**, Speech-responsive electrodes across 20 participants primarily localized to the STG in native (top) and foreign (bottom) speech conditions across hemispheres. Electrode size indicates magnitude of the peak HFA averaged sentence response. Inset histograms show electrode counts across native and foreign speech in each anatomical region. **f**, Acoustic–phonetic TRF weights across three example electrodes show strong correlations between native (top) and foreign (bottom) speech conditions. Different electrodes show tuning to distinct acoustic–phonetic features common to both languages. **g**, TRF weights across speech-responsive electrodes for native (top) and foreign (bottom) speech are highly correlated (Pearson *r*(1,992) = 0.86, *P* < 0.001). Top, columns (electrodes) ordered by the acoustic–phonetic feature showing the maximal weight. Bottom, columns ordered identical to the top for visual comparison. **h**, Distribution of TRF native–foreign weight correlations across electrodes (purple) is higher compared to the non-parametric permuted distribution (grey). Black vertical line indicates the 95th percentile in the permuted distribution. Corr, correlation; LH, left hemisphere; middletemp, middle temporal gyrus; parsoperc, pars opercularis; parstriang, pars triangularis; postcent, postcentral gyrus; precent, precentral gyrus; RH, right hemisphere; superiortemp, superior temporal gyrus; supramarg, supramarginal gyrus; surp, surprisal.

To address this question, we report on a rare dataset collected over the span of 10 years in which we leveraged high-density electrocorticography (ECoG) recordings from a cohort of Spanish, English and Mandarin monolingual speakers passively listening to naturally spoken sentences in their native language and a foreign language (Fig. 1a). The high spatial and temporal resolution of direct cortical surface ECoG enabled us to ask not only about similarities and differences in neural populations activated by native and unfamiliar foreign languages but also much more specifically about how transient speech features are encoded depending on language experience.

We found that the overall magnitude of neural activity in the bilateral STG is similar for native and foreign languages, driven by the largely conserved encoding of acoustic–phonetic speech features. Given this result, we hypothesized that neural signatures present in ECoG recordings of known language processing might be realized at a different representational level: namely, in the enhanced encoding of sequences of acoustic–phonetic features that form larger, language-specific compositional structures, such as syllables and words. Indeed, we found that neural populations more robustly encoded the sequential structure of listeners’ native language, including identifying where individual words begin and end in natural speech. In a separate cohort of Spanish–English bilingual participants, we found that neural populations encoded phoneme sequence and word-level information for both familiar languages, demonstrating experience-dependent mechanisms for speech processing across multiple languages. Finally, in participants with varying degrees of proficiency with English, we found that proficiency modulated the extent to which word-level information in speech could be neurally decoded. We propose that these results support a neurobiological model of human speech processing wherein STG neural populations perform dynamic integration of shared acoustic–phonetic and language-specific phoneme-sequence and word-level information during native language listening.

## Shared phonetic encoding in native and foreign speech

**Extended Data Fig. 1:**
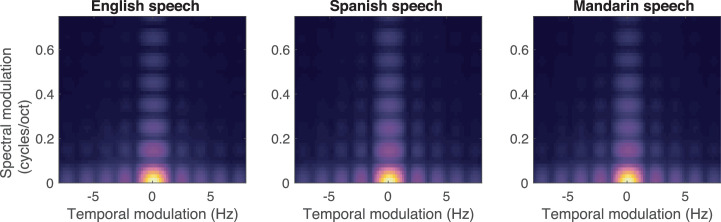
Similar modulation spectrum across speech corpora. Highly similar modulation spectra (qualitative) for English speech stimuli (TIMIT), Spanish speech stimuli (DIMEx), and Mandarin speech stimuli (ASCCD).

To understand how language knowledge affects neural responses to natural speech, we recorded high-density ECoG from participants with diverse language backgrounds undergoing intracranial monitoring for epilepsy while they listened to spoken sentences. Each participant heard sentences in both their known native language and an unknown foreign language (Fig. 1a and Extended Data Fig. 1), allowing us to directly compare activity in the same neural populations.

Because our research question focuses on cross-linguistic phonology rather than morphology, syntax or semantics, we used as stimuli read sentences that were designed to comprehensively span each language’s phonemic inventory^30–32^. We collected ECoG recordings from 20 monolingual participants who spoke Spanish, English or Mandarin (14 left hemisphere, 11 female, 9 Spanish speakers, 8 English speakers and 3 Mandarin speakers), which are three of the most common languages in the world, each spoken by more than half a billion people and originating from two different language families (Indo-European and Sino-Tibetan). Although some participants had received exposure to other languages, they all reported little to no proficiency in the foreign language (Extended Data Table 1) and that they were unable to comprehend when presented with foreign speech. All participants listened to English speech (mean across participants = 20.44 min), Spanish speakers also listened to Spanish (mean = 20.80 min), Mandarin speakers also listened to Mandarin (mean = 23.68 min), and English speakers also listened to Spanish and/or Mandarin (Fig. 1a).

**Extended Data Fig. 2:**
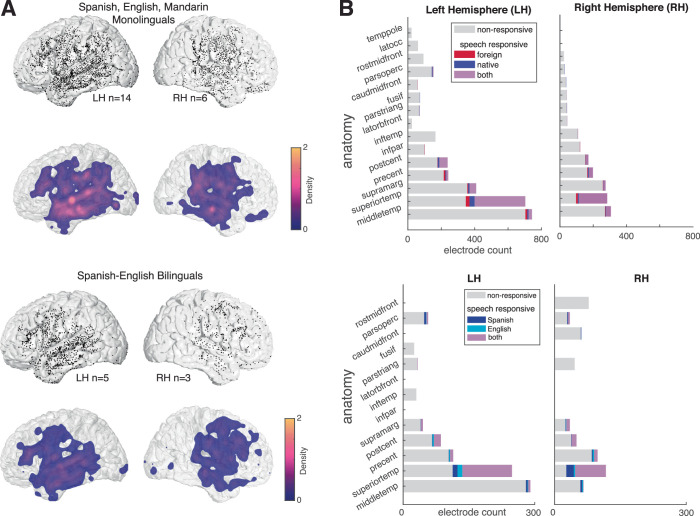
High density lateral surface coverage of the human temporal lobe in English, Spanish, Mandarin speakers and Spanish-English bilinguals. A. Wide and dense coverage of the superior temporal lobe across left and right hemispheres across participants. Anatomical location (MNI) of all electrodes plotted prior to subselecting for speech responsive electrodes. B. Highest counts of electrodes in the left and right hemisphere superior temporal and middle temporal cortices bilaterally. Highest counts of speech-responsive electrodes in superior-temporal cortex. In Spanish, English and Mandarin monolinguals, a majority of speech-responsive electrodes respond to both foreign and native speech, while a minority of electrodes show selective responses to either foreign or native speech. Similarly, in Spanish-English bilinguals, a majority of speech-responsive electrodes respond to both English and Spanish speech.

First, we asked whether the same neural populations respond to both native and foreign speech. We identified 883 electrodes with significantly greater activity during speech than silence in any language condition (high-frequency activity (HFA), 70–150 Hz; see Methods for details and Extended Data Fig. 2 for coverage). An example from a Spanish listener illustrates that the same electrodes were responsive to both native and foreign speech, particularly in the STG (Fig. 1b). Furthermore, the average response dynamics of these electrodes were highly correlated between language conditions (Pearson *r*(250) = 0.98, *P* < 0.001; Fig. 1c). These examples demonstrate that in an individual participant, the same neural populations are active in response to speech regardless of language knowledge.

**Extended Data Fig. 3:**
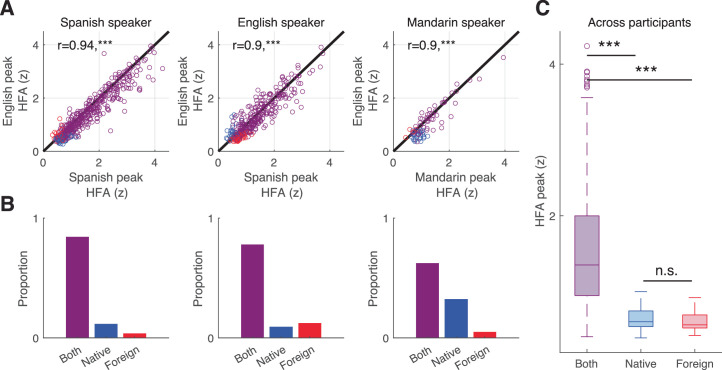
Similar peak HFA response across native and foreign speech conditions. A. Across Spanish, English, and Mandarin speakers, a majority of electrodes are responsive to both native and foreign speech conditions (purple) as opposed to only native (blue) or only foreign (red) speech. Each scatter point corresponds to a single electrode. HFA peak response is calculated as the peak magnitude of the average response across all sentences in the same language. Average HFA peak magnitudes are correlated across speech conditions (Pearson r(472) = 0.94; r(334) = 0.9; r(77) = 0.9, ***p < 0.001 for all participant groups). B. Spanish, English, and Mandarin speakers show no consistent bias in the proportion of electrodes that are only responsive for only either native or foreign speech conditions (blue, red bars). C. Speech responsive electrodes to one language but not the other showed significantly smaller HFA amplitude responses. Electrodes responsive to only foreign or native speech showed no consistent bias in HFA amplitude and no consistent anatomical location (data not shown). Two-sided Wilcoxon rank sum test (n = [705; 113; 65]) determined significance between the distribution of HFA peak magnitudes across groups (***p < 0.001). Box plots show the maximum and minimum values (whiskers), median (center line) and the 25th to 75th percentiles (box limits).

Across all participants, most speech-responsive electrodes were located in the STG, with further responses in the middle temporal, postcentral, precentral and supramarginal gyrus (Fig. 1e). Electrodes with the strongest speech activity were active in response to both native and foreign speech (Extended Data Fig. 3), and 79.84% of electrodes (82.20% median, 10.70% s.d. across participants) showed significant responses to both languages. Single-electrode HFA peaks were not consistently higher, nor was the number of active electrodes significantly more when participants listened to native speech (Extended Data Fig. 3). These results demonstrate a highly overlapping area of cortex activated in response to native and foreign speech^19^.

We next asked whether these neural populations, which were largely active in response to both languages, also encoded the same information, independent of whether the presented language was comprehensible to the listener. All spoken languages consist of the same broad classes of acoustic–phonetic features, which describe short segments such as vowels or consonants^2,3,33^, and therefore neural tuning to these broad classes may be conserved across native and foreign speech. However, Spanish, English and Mandarin phonology differ systematically (for example, in how phonetic features are used to distinguish segments^34–36^), which may give rise to language-specific or selective neural tuning to certain phonetic features.

We evaluated whether language experience affects encoding of acoustic–phonetic features in natural speech. Specifically, we asked, in cases where an acoustic–phonetic feature is present across languages, is it neurally encoded in the same way? To address this question, we fit a temporal receptive field (TRF; with acoustic–phonetic features)^37^ to each language separately and asked the extent to which acoustic–phonetic feature tuning was the same across native and foreign speech. We compared electrode feature tuning across languages by correlating the weights from TRF models that had been fit on each language separately.

We identified electrodes with significant model fits and tuning to specific acoustic–phonetic features and observed that their response patterns were highly correlated across languages. For three example electrodes, tuning to vowel formants, changes in the speech amplitude envelope (peakRate^38^) and fricative manner of articulation showed similar feature weights in native and foreign speech (Fig. 1f). Across electrodes with significant TRF model fits in both languages (*R*^2^ > 0.1), feature weights across languages were highly correlated (Pearson *r*(1992) = 0.86, *P* < 0.001; Fig. 1g).

**Extended Data Fig. 4:**
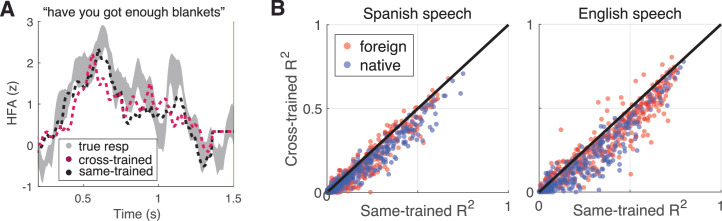
Same and Cross-language Model Predictions. A. Highly similar predicted neural response to an example English sentence from a model trained on another language (Spanish) as opposed to the same language (English). Dashed lines indicate a TRF predicted response. B. Cross-language TRF prediction performance (e.g. training on Spanish speech, testing on English speech) is correlated with same-language prediction TRF performance across both Spanish (Pearson r(883) = 0.96, ***p < 0.001) and English speech (Pearson r(883) = 0.94, ***p < 0.001).

Overall, we found that 155 of 166 electrodes (93.37%) had TRF feature weight profiles correlated above chance (*P* < 0.05 compared to uncorrected non-parametric permuted distribution generated from shuffling TRF weight labels; Fig. 1h). We further tested this by training a TRF model in the native speech condition and testing in the foreign speech condition or vice versa. We found that this cross-training still produced significantly correlated model predictions (Extended Data Fig. 4). These results demonstrate that in addition to the same brain regions being active in response to native and foreign languages, acoustic–phonetic speech feature tuning in local neural populations is largely conserved, indicating that these populations encode the shared auditory content relevant for general speech processing.

Finally, we considered the fact that languages systematically differ from one another in their specific inventory of speech sounds and that language-specific inventories influence speech sound perception, such as categorical perception^39–41^. It is therefore possible that despite the high resolution of the neural data and the sensitivity of TRF-encoding models, neural populations could encode more subtle, categorical differences in phonology between languages^42^. Although natural speech is not ideal for testing these differences because of its inherent multivariate, complex nature, we examined two specific examples of speech sound differences across languages to test whether they are prevalent in STG activity: voice onset time (VOT) and vowel formants.

**Extended Data Fig. 5:**
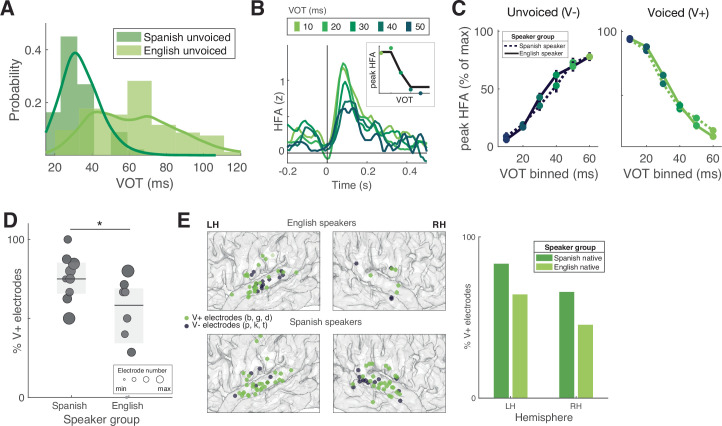
Voice Onset Time Encoding Across Languages. A. VOT values of unvoiced stop-consonants in Spanish (dark green) and English (light green) show systematic differences in duration. This systematic difference along with prior research on the neural encoding of voice onset time (VOT)^26^ suggest that language experience may influence how sounds are processed in the STG. B. Electrodes are sensitive to different VOT values (0:10:60 ms). The top-right inset shows the VOT tuning function for this electrode, where each scatter point corresponds to the peak HFA response to the VOT bin. C. Consistent with prior research, average single electrode tuning curves either showed positive (V-, preferring unvoiced) or negative (V + , preferring voiced) tuning direction. We found no significant difference between tuning curves across VOT sensitive electrodes in Spanish and English speakers (two-tailed Wilcoxon rank sum test, V + n = [57, 32], V- n = [20, 21]). Error bars indicate standard error of the mean across electrodes within each VOT bin for each speaker group. D. Proportion of V+ electrodes was significantly higher (p < 0.05) in Spanish speakers as opposed to English speakers. Size of each scatter corresponds to the number of electrodes within each participant that show significant tuning for VOT. P-value corresponds to language background in a linear mixed effect model (t(128) = 2.11, SE = 0.0176, p = 0.0367). Box plots show the median (center line) and the 25th to 75th percentiles (box limits). E. Intermixed V+ and V- electrodes in English and Spanish speakers. Both left and right hemispheres showed a higher proportion of V+ electrodes in Spanish speakers, suggesting that language experience effects may arise at the population level^25^.

**Extended Data Fig. 6:**
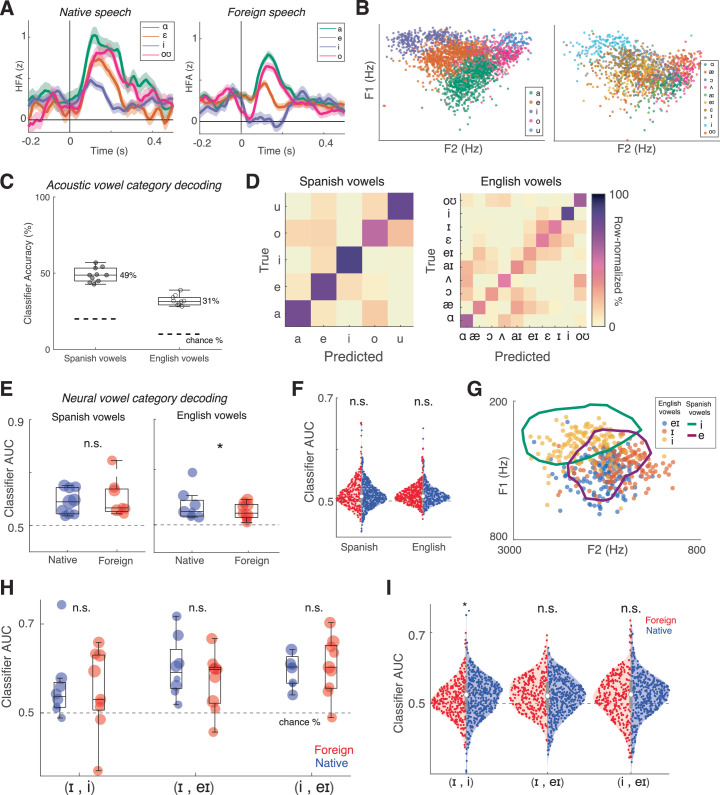
Vowel Category Encoding Across Languages. A. Example electrode response to approximately equivalent vowel categories across languages (English, Spanish) shows qualitatively similar response patterns in native and foreign speech conditions. Shaded patches indicate standard error of the mean HFA response across vowels. B. Formant frequencies (Hz) from each vowel instance are shown in the scatter and colored by the vowel category they belong to. Spanish (left) and English (right) vowel spaces include a different number of vowels, and contain different formant space distributions for each category. C. Acoustic vowel category decoding (10-fold linear discriminant analysis) accuracy using the formant values of vowel sounds across English and Spanish speech corpora. Each scatter point represents 1/10 validation folds. Chance accuracy was calculated based on the number of vowel categories included from the particular language (Spanish: 1/5, English 1/10). Vowel categories with more than 100 instances in the speech corpus were included. Box plots show the maximum and minimum values (whiskers), median (center line) and the 25th to 75th percentiles (box limits). D. Confusion matrix resulting from acoustic vowel category decoding exhibits a clear diagonal, with off-diagonal confusions reflecting overlap in F1/F2 space (e.g. /a/ and /e/). E. Single participant neural vowel category decoding (10-fold linear discriminant analysis; each scatter corresponds to a single participant) separated by speech condition shows no significant difference in Spanish (LME t(168) = 0.49, SE = 0.0198, p = 0.63, see “Methods” for formula) and a weak effect in English (LME t(168) = 1.98, SE = 0.0095, *p = 0.049). Box plots show the maximum and minimum values (whiskers), median (center line) and the 25th to 75th percentiles (box limits). F. Single electrode neural vowel category decoding (10-fold linear discriminant analysis; each scatter corresponds to a single electrode) separated by language shows no significant effect of speech condition (two-sided Wilcoxon rank sum test Spanish n = [453, 302], p = 0.23); English n = [292, 416], p = 0.86). G. Vowel formants (F1, F2) for instances of “i”, “I”, and “ei” in English are subsumed by only two vowel categories in Spanish, “i” and “e”. 95th percentile contours for Spanish vowel formants shown as colored lines. H. Pairwise neural decoding AUC (10-fold logistic regression) for three English vowel categories, “i”, “I”, and “ei” shows no significant difference between participant groups (LME t(168) = −0.029, p = 0.98; t(134) = −0.87, p = 0.38; t(134) = 0.84, p = 0.40, see “Methods” for formula). Each scatter point corresponds to the mean AUC for a single participant (colored by speech condition). Box plots show the maximum and minimum values (whiskers), median (center line) and the 25th to 75th percentiles (box limits). I. Pairwise neural decoding AUC (10-fold logistic regression) for the same English vowel categories, shows no significant difference between single electrodes across participant groups. Each scatter point corresponds to the mean AUC for a single electrode (two-sided Wilcoxon rank sum test n = [416, 292], p = 0.02; p = 0.08; p = 0.24).

Spanish and English differ systematically in how the acoustic cue of VOT is used to determine phoneme category membership for plosive sounds^36,39^. We found a small but significant effect of native language on the distribution of electrodes tuned to VOT in the predicted direction (more electrodes tuned to voiced plosives in Spanish speakers) but no clear difference in how single electrodes encode plosives (Extended Data Fig. 5). In addition to VOT, Spanish and English differ in the number and acoustic realization of language-specific vowel categories^43^. In line with our VOT results, we found no consistent, significant differences between the neural encoding of vowels in English and Spanish speakers (Extended Data Fig. 6). These results indicate that although categorical differences between phonemes can be affected by language experience (perhaps best studied with stimuli that parametrically dissociate acoustic cues, such as in ref. ^35^), the dominant pattern in STG when presented with natural speech is one of shared acoustic–phonetic representation across languages where categorical effects are overridden by the availability of other cues.

Together, these results indicate that the same STG neural populations respond to speech regardless of whether the listener is familiar with the language presented. Particularly in high-level auditory areas like the STG, neural populations encode largely the same acoustic–phonetic speech content, reflecting common vocal tract articulator movements and the resulting shared phonological structure of different languages.

## Language experience enhances word encoding

Given that the brain represents individual speech sounds similarly regardless of language experience (Fig. 1), we hypothesized that experience is reflected more strongly in listeners’ ability to compose these sounds into phonological sequences and words. This hypothesis can be intuited by the fact that when we hear someone speaking an unfamiliar language, we may be able to tell broadly which consonants and vowels we hear, but we find it substantially more challenging to identify where one word ends and another begins. This is because natural speech does not have reliable cues to word boundaries^44–46^ and different languages such as Spanish, English and Mandarin are highly distinct at the level of speech sound sequences and words, specifically with respect to phonotactic and timing-related differences^34,47^. As a result, we were able to specifically test the hypothesis that when listening to one’s native language, compared to an unfamiliar foreign language, language experience supports the neural encoding of phonological structure^48^, which is in turn used to segment the continuous speech stream into a series of auditory objects like words.

**Fig. 2:**
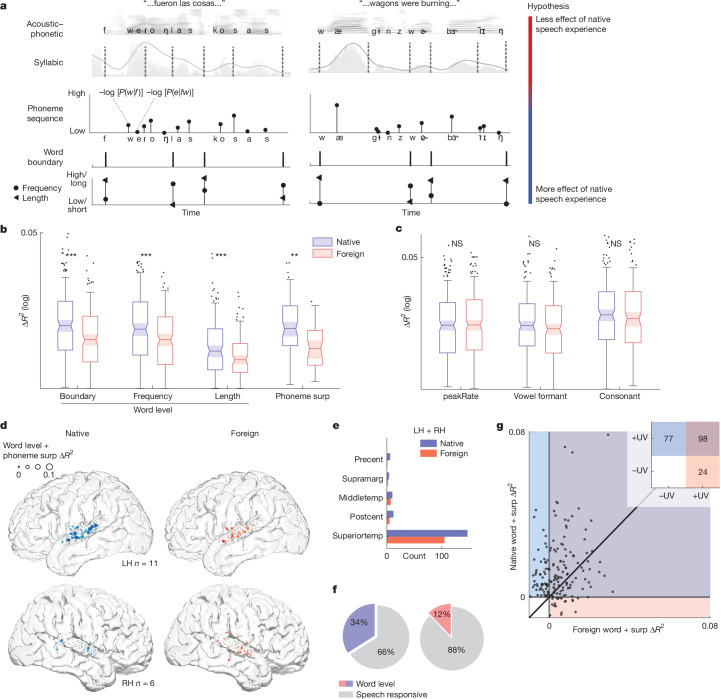
Enhanced encoding of word-level and phoneme-surprisal features in native speech. **a**, Spanish (left) and English (right) sentences annotated with phonemes, syllable boundaries, phoneme surprisal, word boundaries, frequencies and lengths. We hypothesized that neural encoding of speech representations requires increasing language experience (colour bar at right). **b**, Across electrodes, the variance in the neural response uniquely explained by word-level features is significantly higher in native versus foreign speech (LME model boundary ***P < 0.001, frequency ***P < 0.001, length ***P < 0.001; see Methods for formula) and phoneme surprisal (LME **P = 0.0012). Box plots show non-outlier maximum and minimum values (whiskers), median (centre line) and lower and upper quartiles (box limits) corresponding to electrodes surpassing a significance threshold for model *R*^2^ > 0.05 and Δ*R*^2^ > 0.001 (boundary *n* = 246, frequency *n* = 256, length *n* = 126). **c**, Unique variance for acoustic–phonetic features does not significantly differ in native versus foreign speech (LME peakRate *P* = 0.41, formant *P* = 0.60, consonant *P* = 0.31; see Methods for formula). Box plots show non-outlier maximum and minimum values (whiskers), median (centre line) and lower and upper quartiles (box limits) corresponding to electrodes surpassing a significance threshold for model *R*^2^ > 0.05 and Δ*R*^2^ > 0.001 (peakRate *n* = 502; formant *n* = 316; consonant *n* = 270). **d**, Electrodes with positive unique variance for word and phoneme-surprisal features primarily located in mid-STG; native (left) and foreign (right) speech. Electrode size indicates unique variance magnitude. Black scatters correspond to speech-responsive electrodes. **e**, Electrode counts with significant word and phoneme-surprisal feature unique variance (permutation test versus shuffled distribution) across native and foreign speech in each anatomically defined brain region. **f**, Proportion of speech-responsive electrodes with significant word and phoneme-surprisal feature unique variance (permutation test versus shuffled distribution) for speech conditions across hemispheres; native (left) and foreign (right) speech. **g**, Ninety-eight electrodes show positive unique variance for word and phoneme surprisal in both native and foreign languages; unique variance for word and phoneme surprisal is not significantly correlated across languages (Spearman *r*(98) = 0.16, *P* = 0.12). Inset plot shows electrode count in each quadrant of the scatter plot. NS, not significant.

To test this hypothesis, we fit TRF models with the same acoustic–phonetic features as in Fig. 1 and other features that we predicted would be relevant for identifying the phonological structure of words in continuous speech: word boundary, word length^49,50^, word frequency^51,52^ and phoneme surprisal (negative log probability^53^; for a full predictor list, see Methods; Fig. 2a). We limited the following analyses to the Spanish- and English-speaking participants to perform a fully crossed comparison in which all participants listened to the same two languages (English and Spanish).

To evaluate the specific encoding of word-level features and phoneme surprisal independent of acoustic–phonetic tuning, we used unique *R*^2^ as a measure of explained variance^12,54^. Across speech-responsive electrodes, word boundary, length and frequency explained significantly greater unique variance for native compared to foreign speech (linear mixed effects (LME) word boundary *t*(243) = 4.00, standard error (s.e.) = 0.0008, *P* < 0.001; length *t*(123) = 3.65, s.e. = 0.0006, *P* < 0.001); frequency *t*(253) = 5.18, s.e. = 0.0006, *P* < 0.001); Fig. 2b). We also found that phoneme surprisal explained significantly greater unique variance in native compared to foreign speech (*t*(29) = 3.58, s.e. = 0.0017, *P* = 0.0012; Fig. 2b), indicating that response to sublexical sequence information is also dependent on language experience. These language-experience-dependent effects were in contrast to no differences for acoustic–phonetic feature encoding (Fig. 2c; all *P* > 0.05), consistent with the analysis of acoustic–phonetic feature weights (Fig. 1e–g). As a further control, we tested differences in unique variance explained by pitch (absolute pitch and relative pitch^55^) and the continuous speech envelope, both of which showed no significant differences (pitch *t*(175) = 0.55, s.e. = 0.0027, *P* = 0.58; envelope *t*(473) = 1.191, s.e. = 0.0009, *P* = 0.23). This result establishes that a key neural difference between hearing one’s native versus a foreign language is in how the phonological structure of speech is encoded towards the goal of perceiving words.

**Extended Data Fig. 7:**
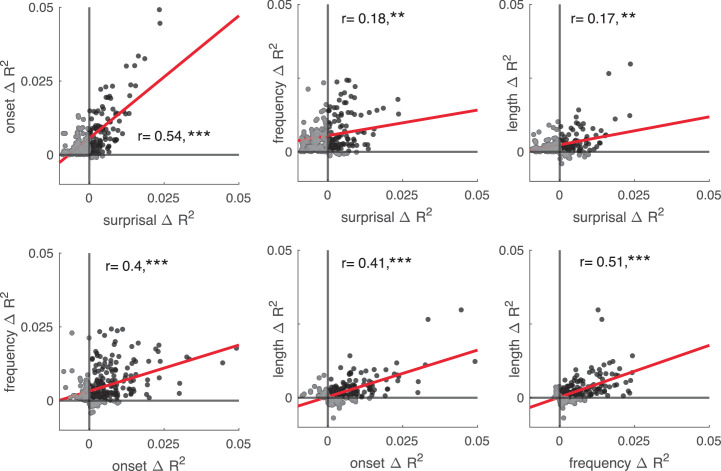
Comparing Word and Phoneme Surprisal Unique Variances. Significant correlations between positive unique variance values for word onset vs. surprisal (Pearson r(257) = 0.54, ***p < 0.001), frequency vs. surprisal (Pearson r(268) = 0.18, **p = 0.0033), length vs. surprisal (Pearson r(250) = 0.17, **p = 0.009), word onset vs. frequency (r(303) = 0.4, ***p < 0.001), word onset vs. length (Pearson r(301) = 0.41, ***p < 0.001), and length vs. frequency (r(293) = 0.51, ***p < 0.001) in the native speech condition.

**Extended Data Fig. 8:**
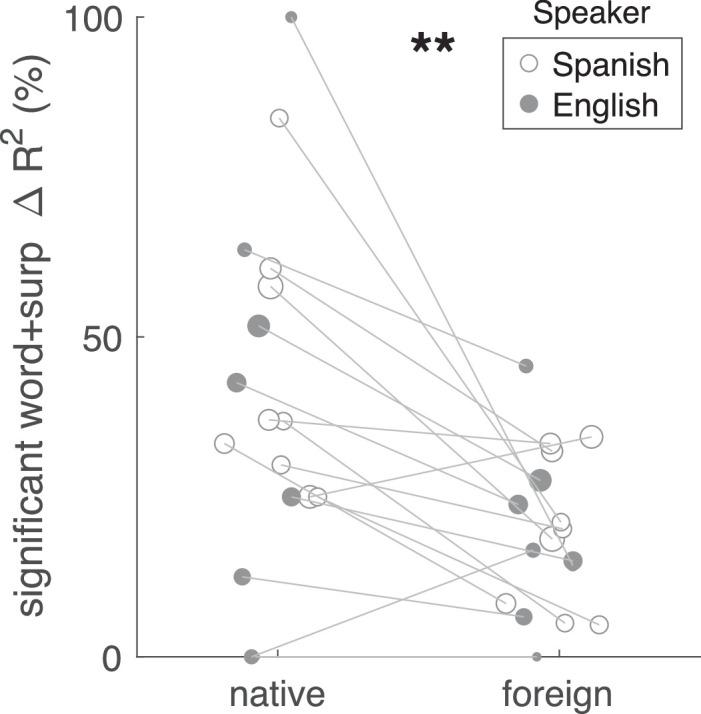
Proportion of Word-level Electrodes Across Conditions. Proportion of electrodes with significant word and phoneme surprisal unique variance for single participants across native and foreign speech conditions. Significance determined by a 1-tailed paired Wilcoxon signed-rank test (n = 17, **p = 0.0012).

Next, we asked where on the lateral brain surface language-experience-dependent information was predominantly encoded (we combined the word-level and phoneme-surprisal features because encoding of each is strongly correlated; Extended Data Fig. 7). We found that electrodes showing significant unique variance for these features were primarily located in the bilateral mid-STG (Fig. 2d,e). These electrodes made up  34% of the speech-responsive electrodes identified in Fig. 1 in the native speech condition and 12% of the speech-responsive electrodes in the foreign speech condition (Fig. 2f). Across single participants, the percentage of electrodes significantly encoding word-level and phoneme-surprisal features was significantly higher in the native language (Extended Data Fig. 8; signed-rank test *n* = 17, *P* = 0.0012).

If encoding of phonological structure is largely dependent on language experience, it is perhaps surprising that we find any significant encoding of this information in foreign speech. To understand the extent to which the encoding of phonological structure in foreign speech reflects the same processes as in the native language, we compared the unique variance for word-level and phoneme-surprisal features across languages. A minority of electrodes encoded phonological structure only in the foreign language (lower-right quadrant), potentially implicating a learning signal or tuning to new sound structure^56^. Overall, encoding of phonological structure was significantly stronger in the native language (word level and phoneme surprisal *t*(179) = 4.77, s.e. = 0.0018, *P* < 0.001), including for electrodes that had significant effects in both native and foreign languages (Fig. 2g). Furthermore, the pattern of unique variance was not significantly correlated between languages (Fig. 2g; Spearman *r*(98) = 0.16, *P* = 0.12). Together, this indicates that although the brain may leverage native-like processing to attempt to parse foreign speech, the ability to encode phonological sequence information and words is significantly stronger in one’s native language.

## STG segments words in native speech

**Fig. 3:**
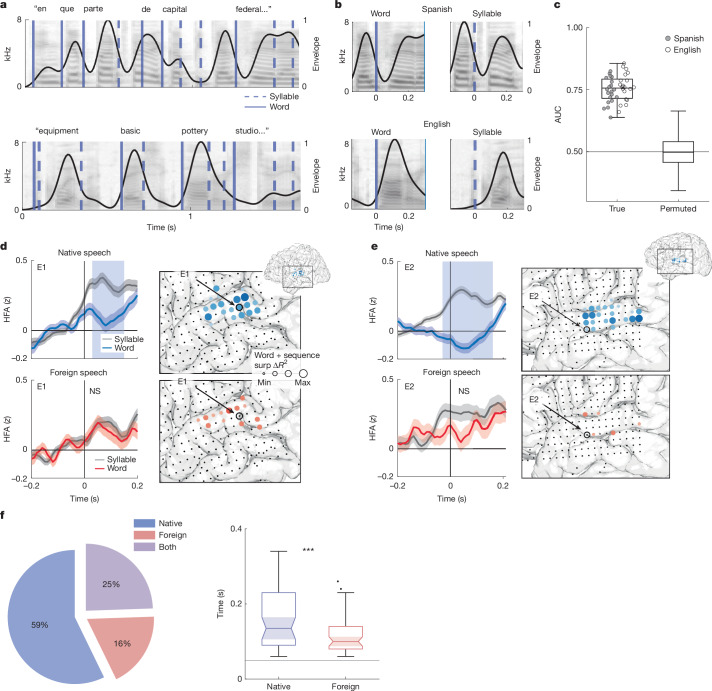
Enhanced discrimination between word and syllable boundaries in native speech. **a**, Spectrogram and speech envelope for example Spanish (top) and English (bottom) sentence stimuli. Word and syllable boundaries marked in solid and dashed lines, respectively. **b**, Spectrogram and speech envelope for example word and syllable boundaries are closely matched to one another in Spanish (top) and English (bottom) but differ across languages. Example word and syllable boundaries marked as in **a**. **c**, Acoustic classification of word boundaries using the spectrogram across English and Spanish speech. Each scatter point represents 1/20 folds. Permuted distribution median AUC of 0.50 (25 permutations with 20 folds each for each language). Box plots show maximum and minimum values (whiskers), median (centre line) and 25th to 75th percentiles (box limits). **d**, Left, example electrode (E1) from an English listener differentiates word and syllable boundaries in native (top) but not in foreign (bottom) speech. Rectangular patches indicate time points with a significant difference (per-time-point two-sided ANOVA *P* < 0.01 with Bonferroni correction). Continuous shaded patches indicate standard error of the mean HFA response across boundaries. Right, anatomical location of E1 and the unique variance for word and phoneme-surprisal features in the example participant. **e**, Left, example electrode (E2) from a Spanish listener differentiates word and syllable boundaries in native (top) but not in foreign (bottom) speech. Rectangular patches indicate time points with a significant difference (per-time-point two-sided ANOVA *P* < 0.01 with Bonferroni correction). Continuous shaded patches indicate standard error of the mean HFA response across boundaries. Right, anatomical location of E2 and unique variance for word and phoneme-surprisal features in the example participant. **f**, Word versus syllable boundary discrimination is significant in more electrodes (left) and for a longer duration of the neural response (right) in native versus foreign speech (LME ***P < 0.001; see Methods for formula).

When we hear speech in a foreign language, a salient challenge for listeners is identifying when individual words begin and end (word segmentation)^44,57^. This is in part due to the fact that there are no reliable acoustic cues to word boundaries. Even prominent acoustic cues to word boundaries in English, like sharp increases in the speech envelope at word onset^58–60^, are unreliable because they can also occur at syllable boundaries (Fig. 3a,b). Indeed, a decoder trained to classify word boundaries using 0.2 s of the spectrogram around the boundary performs with a mean accuracy of 0.72 (median area under the receiving operating curve (AUC) = 0.76 logistic regression, 20-fold cross-validated), meaning that more than one in four words are incorrectly segmented on the basis of acoustic cues alone (Fig. 3c).

Given that listeners show enhanced representation of linguistic features relevant for words in their native language (word boundary, length and phoneme-surprisal features; Fig. 2b), we hypothesized that neural activity would also be better able to discriminate between word and syllable boundaries in one’s native compared to a foreign language. We first compared neural responses aligned to boundary events in individual electrodes. We found electrodes in STG that showed differential responses to word and syllable boundaries only for native speech for both Spanish and English speakers (Fig. 3d,e). Specifically, in native speech, both example electrodes show a negative deflection in amplitude immediately after a word boundary as compared to the positive peak in amplitude at the syllable boundary^61^. Of the electrodes that discriminated between boundary events (50 ms of contiguous significant difference, *P* < 0.01 with Bonferroni correction), most did so only in native speech (Fig. 3f; 59%). Further, for electrodes that discriminated between boundary events in both languages (Fig. 3f; 25%), discrimination occurred for significantly longer in native speech (Fig. 3f; *t*(485) = 9.987, s.e. = 0.6955, *P* < 0.001). These results indicate that language experience enhances the brain’s ability to discriminate between word and syllable boundaries in continuous speech, leveraging knowledge about the phonological structure of words to overcome ambiguous acoustic cues.

**Fig. 4:**
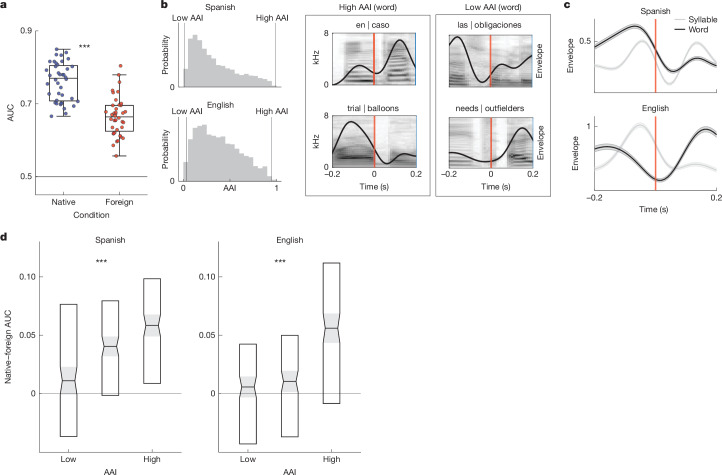
Enhanced neural word-boundary decoding in native speech. **a**, Neural classification of word boundaries is significantly better in native versus foreign speech (each scatter point corresponds to the AUC of 1/20 folds combined across English and Spanish; one-tailed two-sample *t*-test ***P < 0.001). Box plots show the maximum and minimum values (whiskers), median (centre line) and 25th to 75th percentiles (box limits). **b**, Left, AAIs span a similar range in Spanish (top) and English (bottom). AAI values were derived from an acoustic word-boundary decoder trained separately on Spanish and English speech stimuli. Right, spectrogram and envelope of high- and low-AAI examples, such as ‘trial | balloons’ and ‘needs | outfielders’, which reflect the acoustics of highly unlikely and likely transitions at a word boundary in American English, respectively. **c**, Low-AAI examples reflect the language-specific acoustic properties of word and syllable boundaries in Spanish (top) and English (bottom) as demonstrated by the average speech envelope. This resemblance can be seen by comparing low-AAI examples in **b** to the average envelope aligned to the word boundary (black trace). Shaded patches around each trace are the standard error of the envelope across boundary events in the given language. **d**, Native language sensitivity to word boundaries is enhanced for ambiguous word boundaries; Spanish (left) and English (right). The difference between native and foreign neural word-boundary decoding (AUC) increases as AAI increases. Box plots show median (centre line) and 25th to 75th percentiles (box limits), with each distribution corresponding to 225 pairwise AUC difference values generated by random sampling. AUC differences show a significant effect of bin (one-way ANOVA ***P < 0.001 in both languages).

**Extended Data Fig. 9:**
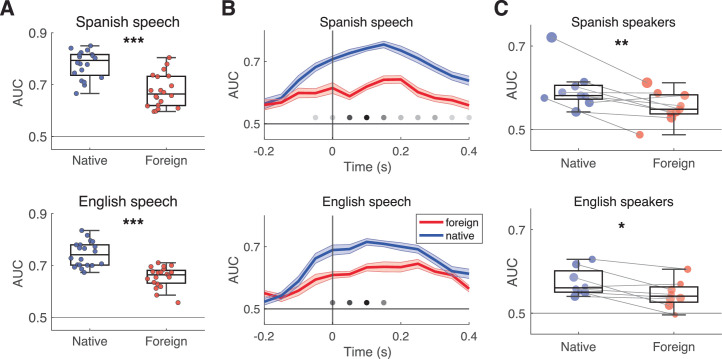
Characterization of Neural Word Boundary Decoding. A. Neural classification of word boundaries is significantly better in native vs. foreign speech (each scatter point corresponds to the AUC of 1/20 folds shown separately for English and Spanish; 1-tailed 2-sample t-test ***p < 0.001). Box plots show the maximum and minimum values (whiskers), median (center line) and the 25th to 75th percentiles (box limits). B. Temporally-resolved neural classification of word boundaries (increments of 0.05 s and 0.2 s before to 0.4 s after boundary, mean across 20 folds) is significantly better in native vs. foreign speech approximately 0.1 s after the boundary event (per-time point two-sided ANOVA p < 0.01 with Bonferroni correction show significant time-points as black scatter points). Continuous shaded patches indicate standard error of the mean across decoding folds for each time point. C. Neural classification of word boundaries is significantly better in native vs. foreign speech when considering single participant decoding performance (each scatter corresponds to median AUC across 20 folds per participant separated by group, size corresponds to the number of electrodes used for classification). P-values derived from a 1-tailed paired Wilcoxon signed-rank test (Spanish speakers n = 9, **p = 0.0098; English speakers n = 8, *p = 0.012). Box plots show the maximum and minimum values (whiskers), median (center line) and the 25th to 75th percentiles (box limits).

To quantify the extent to which neural word segmentation was dependent on language experience, we trained a decoder to use ECoG recordings to classify word and syllable boundaries in each participant group and compared the performance (AUC) across cross-validated folds (0.2 s before to 0.4 s after each boundary; 20-fold cross-validated logistic regression). We found that word-boundary decoding performance was significantly higher in native compared to foreign speech (native AUC = (0.77 ± 0.052); foreign AUC = (0.66 ± 0.053); Fig. 4a). We also found that this effect was consistent in each speech corpus separately and in single participants (Extended Data Fig. 9).

**Extended Data Fig. 10:**
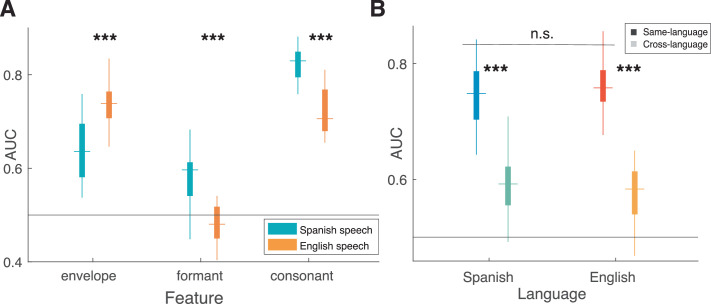
Characterization of the Acoustic Cues to Word Boundary. A. In Spanish and English speech, different acoustic-phonetic features cue word and syllable boundaries. We used three distinct acoustic-phonetic features: continuous envelope, continuous formants, and consonant features around each boundary event to classify word vs. syllable boundary. All features we tested show significantly different AUC across English and Spanish (2-tailed Wilcoxon signed-rank test, for all features n = 20, ***p < 0.001). Box plots show the maximum and minimum values (whiskers), median (center line) and the 25th to 75th percentiles (box limits). B. Acoustic classification of word boundary trained and tested on the spectrogram from the same language shows significantly higher AUC than a classifier trained on one language and tested on the other (2-tailed Wilcoxon signed-rank test, same-language vs. cross-language n = 20, ***p < 0.001; English vs. Spanish n = 20, p = 0.37), suggesting acoustic cues to word boundaries are distinct in Spanish and English. Box plots show the maximum and minimum values (whiskers), median (center line) and the 25th to 75th percentiles (box limits).

Although word segmentation is challenging without language experience, it is still possible for naive listeners to identify some boundaries on the basis of salient acoustic cues^29,62^ (Fig. 4b,c and Extended Data Fig. 10). Therefore, we hypothesized that language experience particularly enhances word-boundary decoding for words with unreliable acoustic cues. We characterized each word boundary according to an acoustic ambiguity index (AAI) that measures the extent to which acoustic cues could be used to classify each instance as a word or within-word syllable boundary (see Methods for details; Fig. 4b). We computed AAIs for Spanish and English separately because acoustic and prosodic cues to word boundaries are language specific^29,63,64^ (Fig. 4c and Extended Data Fig. 10) Critically, in both languages, the envelope of high-AAI word-boundary examples (Fig. 4b) strongly resembles the average envelope aligned to within-word syllable boundaries rather than word boundaries (Fig. 4c). This indicates that if unfamiliar listeners were to use average envelope patterns alone to identify word boundaries, they would incorrectly classify the high-AAI boundary trials.

Next, we binned the continuous AAI metric into three discrete bins and compared native versus foreign neural word-boundary decoder performance (decoders from Fig. 4a). To do this, we randomly sampled trials to generate a distribution of AUC values in each bin and computed the pairwise difference between AUC values across speech conditions (native–foreign). We found that as acoustic cues became more ambiguous (increasing AAI), the difference in AUC between the native and foreign neural decoders increased as well, which we found to be significant using a one-way analysis of variance (ANOVA) (Fig. 4e; Spanish *F*(2, 672) = 12.88, *P* < 0.001; English *F*(2, 672) = 24.91, *P* < 0.001). This result demonstrates that enhanced neural decoding of word boundaries in native speech is probably driven not only by sensitivity to the acoustic–phonetic cues to word boundaries but also by cues that draw on language experience and are not explicit in the acoustic signal.

## STG identifies words across languages

The vast majority of the world’s population is proficient in more than one language. Although it is known that in bilingual individuals, both familiar languages evoke neural activity in the same core set of brain regions^65,66^, it remains unclear what this activity reflects. Here we took advantage of rare opportunities to record from bilingual participants while they listened to continuous speech in both of their familiar languages. We specifically asked the extent to which encoding of both acoustic–phonetic and word-level information is the same for both languages and how these representations differ as a function of second-language proficiency.

**Fig. 5:**
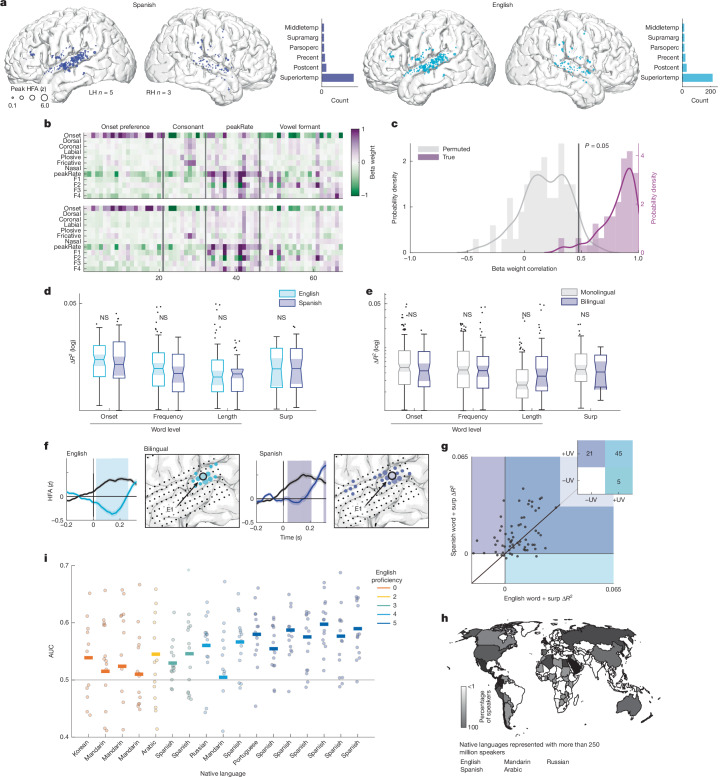
Bilingual listeners show neural encoding of word-level features and phoneme surprisal for both familiar languages. **a**, Speech-responsive electrodes across eight Spanish–English bilingual participants show similar STG localization for Spanish (left) and English (right) speech across hemispheres. Electrode size indicates magnitude of the peak HFA averaged sentence response. Inset histograms show electrode counts across English and Spanish speech in each anatomical region. **b**, TRF weights across speech-responsive electrodes for English (top) and Spanish (bottom) are significantly correlated (Pearson *r*(804) = 0.88, *P* < 0.001). Top, columns (electrodes) ordered by the acoustic–phonetic feature showing the maximal weight. Bottom, columns are ordered identical to top for visual comparison. **c**, Distribution of TRF Spanish–English weight correlations across electrodes (purple) is significantly higher compared to the non-parametric permuted distribution (grey). Black vertical line indicates the 95th percentile in the permuted distribution. **d**, Encoding of word-level and phoneme-surprisal features is not significantly different across Spanish and English (LME speech condition *P* > 0.05; see Methods for formula). **e**, Encoding of word-level and phoneme-surprisal features is not significantly different between bilingual and the native language for monolingual participants (LME speech condition *P* > 0.05; see Methods for formula). **f**, Example electrode from a Spanish–English bilingual participant differentiates word and syllable boundaries in both the English speech condition (left) and Spanish speech (right). **g**, Forty-five electrodes show positive unique variance for word-level and phoneme surprisal in English and Spanish. Unique variance for word-level features across languages is significantly correlated (Spearman *r*(45) = 0.31, *P* = 0.03). Inset plot shows the count of electrodes in each quadrant of the scatter plot. **h**, World map indicating language backgrounds represented in the participant cohort and extent to which these languages are spoken across the world. Maps generated using MathWorks mapping toolbox and information from the World Factbook^105^. **i**, Neural word-boundary classification AUC in English scales with English language proficiency for bilingual speakers of Spanish–English and speakers of diverse languages (LME proficiency *P* < 0.001; see Methods for formula). Each line corresponds to the mean AUC per participant; each scatter point is AUC of 1/15 folds.

First, we focused on recordings from eight Spanish–English bilingual participants (five left hemisphere, three female) who were highly proficient in both languages (see Extended Data Table 3 for language profiles and Extended Data Fig. 2 for coverage). Consistent with the results from participants familiar with only one language (Fig. 1), we found that similar anatomical regions were active regardless of whether bilingual participants listened to English or Spanish, with the highest proportion of electrodes located in the STG (Fig. 5a). Activity in this region could be explained by encoding of acoustic–phonetic speech features (Fig. 5b), which was significantly correlated across languages (64 of 67 electrodes, 95.52%) and had TRF feature weight profiles that were correlated above chance (*P* < 0.05 compared to uncorrected non-parametric permuted distribution generated from shuffling TRF weight labels; Fig. 5c). Thus, in the brain of a bilingual participant, encoding of spectrotemporal speech content in the STG is substantially similar in multiple languages (Fig. 5b,c).

Above, we showed that language knowledge most prominently affects representations of word-level and phoneme-sequence speech information. Because Spanish–English bilingual participants are familiar with the statistics of Spanish and English, we hypothesized that neural activity would reflect encoding of this information in both languages, unlike in the monolingual participants (Fig. 2). Using a LME model, we found that word-boundary, frequency, length and phoneme-surprisal unique variance was not significantly different between the two languages in bilingual participants (Fig. 5d) (boundary *t*(163) = −0.05, s.e. = 0.0009, *P* = 0.96; frequency *t*(187) = −1.93, s.e. = 0.0008, *P* = 0.055; length *t*(177) = −1.20, s.e. = 0.0008, *P* = 0.23; surprisal *t*(73) = 0.76, s.e. = 0.0011, *P* = 0.45). Furthermore, encoding of word-level and phoneme-surprisal features was not significantly different in bilingual participants as compared to the native language condition for monolingual participants (Fig. 4e) (boundary *t*(321) = −1.28, s.e. = 0.0010, *P* = 0.20; frequency *t*(346) = 0.23, s.e. = 0.0010, *P* = 0.82; length *t*(318) = 1.37, s.e. = 0.0072, *P* = 0.17; surprisal *t*(144) = −1.09, s.e. = 0.0010, *P* = 0.28). Together, these results demonstrate that language experience—even for multiple languages in a single brain—modulates the extent to which word-level and phoneme-surprisal features affect neural representations in the temporal lobe.

Bilingual participants provide a unique opportunity to determine whether word-level and phoneme-surprisal encoding electrodes are language specific—that is, whether electrodes encode these features for only one of the two familiar languages. In contrast to monolingual participants (Fig. 3), in bilingual participants, we found STG electrodes that strongly differentiated word and syllable boundaries in both Spanish and English (Fig. 5f). To test for language specificity across electrodes, we compared the corresponding unique variances for word-level and phoneme-surprisal features across Spanish and English. We found that a large proportion of electrodes encoded word-level and surprisal features for both English and Spanish, similar to results reported for monolingual participants in Fig. 2. However, unlike the monolingual participants, the unique variances across languages were significantly correlated between languages (Fig. 5g; Spearman *r*(45) = 0.31, *P* = 0.03). This result demonstrates that word and phonological sequence information can be represented in the same populations across languages and implicates a shared mechanism for encoding phonological structure across languages in the bilingual brain.

Finally, although the key results thus far have been demonstrated with three languages that represent some of the linguistic diversity that exists across countries and cultures (English, Spanish and Mandarin), there are many other languages and language families acquired as native languages throughout the world. Over more than a decade, we sampled some of these languages by collecting data from participants who were speakers of Russian, Arabic and Korean (representing Indo-European, Semitic and Koreanic language families; Fig. 5h). Furthermore, these participants had varying degrees of self-reported English proficiency (see Extended Data Table 4 for language profiles), allowing us to test the effects of experience across a wider array of known languages that differ from English to varying degrees. Given that the ability to identify word boundaries is one of the key phonological markers of language knowledge^67^, we asked the extent to which neural English word-boundary decoding accuracy is graded as a function of English proficiency across these participants. Using a LME model, we found that AUC was significantly correlated with English proficiency (Fig. 5i; *t*(253) = 4.58, s.e. = 0.002, *P* < 0.001), indicating that the brain’s ability to extract word-level structure from continuous speech depends on listeners’ overall knowledge of the language. Although these results arise from limited data, the fact that proficiency rather than typology of the native language (Russian, Korean and so on) predicts neural word-boundary decoding in English indicates that neural encoding of word boundaries may be a general speech processing mechanism that arises independently for each language that an individual learns.

## Discussion

One of the remarkable attributes of the human brain is the ability to master the complexities of spoken language in just the first few years of life. Yet this skill does not transfer to all languages—we only understand languages to which we have been sufficiently exposed. This fact is underscored when we listen to a foreign language; although we may be able to identify individual speech sounds that are similar to the sounds of languages we know, it is substantially more challenging to parse the continuous input into larger chunks like words. To date, it has been unclear what drives this gap in our perception of native and foreign languages.

In this study, we leveraged high-resolution direct brain recordings to provide a detailed picture of the neural processes that reflect shared auditory phonological processes and those that are influenced by language experience. Owing to shared human anatomy, the same fundamental articulatory features make up speech in all languages^68^: features the human brain is readily able to perceive. However, with exposure that begins before birth, the brain becomes exquisitely tuned to the fine-grained, specific characteristics of the native language, including the specific inventory of speech sounds used by that language^7,8,69–72^. Throughout development, listeners become sensitive to how these sounds are combined to form the words that make up the language^10,11,73^, which are also specific to each of the world’s 7,000 languages. Here we demonstrate that language experience more strongly affects this latter ability, with enhanced neural encoding of word-level features associated only with the listener’s native language.

Our results showing shared neural representations of speech in the human temporal cortex confirm its role as a processor of fundamental speech features^2^, independent of language experience. One surprising aspect of our results is the finding that experience-dependent neural activity also exists in these brain regions that process both native and foreign language input. This challenges the prevailing view that language processing only happens in a network of cortical regions that are selective for comprehensible speech^24^. Theoretical models of speech processing posit that auditory regions like the STG perform surface-level spectrotemporal extraction before sound input is linguistically transformed in higher-order cortical regions^13,74,75^. Under this view, the STG is a language-agnostic speech processor and therefore discriminates between languages as a consequence of distinct acoustic properties but not language experience of the listener. By contrast, we find evidence that representations of acoustic–phonetic, sublexical and even word-level information are intermixed throughout the STG, with neural populations and representations showing varying levels of sensitivity to language experience. Ultimately, the current results are consistent with a view of the STG as a high-order auditory region, acting as an interface between speech input and linguistic knowledge^3,76,77^.

The present results demonstrate how language knowledge affects neural populations in the temporal lobe that are classically thought to process lower-level acoustic information^13,24^. These findings do not fully disambiguate the extent to which language experience-dependent knowledge is encoded elsewhere in the brain and exerts a top-down modulatory effect on STG^78,79^. However, the fact that the strongest and most prevalent encoding of language-specific word-level information is in the STG indicates that these neural populations and underlying circuitry may be intrinsically sensitive to this type of learned knowledge^3^.

The present study is limited in that we did not sample from all brain regions potentially relevant to language experience. In particular, these results are based on ECoG responses recorded from the lateral surface that did not allow for the examination of the superior temporal plane such as the primary auditory cortex on Heschl’s gyrus, the superior temporal sulcus or subcortical structures that can be structurally and functionally affected by language experience^12,74,80–82^. Previous recordings from Heschl’s gyrus with native English speakers listening to English indicate that these neural populations may be less sensitive to the acoustic–phonetic and word-level features examined here^12^. It also remains to be seen whether our findings extend to learned non-speech sound stimuli, such as music^83–85^.

These findings have implications for understanding how language comprehension abilities arise during development and for explaining the challenges associated with learning new languages later in life. Although the adult brain retains an ability to learn new linguistic information^56,86–89^, achieving native-like proficiency with the phonology of a language after development can be difficult and depends on several different usage factors^90–93^. Our results indicate that this challenge is in part related to learning how to parse sequences of individual speech sounds into language-specific units such as words.

In bilingual participants, neural encoding of language-specific knowledge (English, Spanish) is localized to the same brain regions, the STG, and in some cases even occurs in the same neural populations (Fig. 5g). These findings are consistent with theoretical models that posit a single and highly overlapping brain network in which multiple languages are processed^94–99^. Although language-specific neural activation and impairments post brain injury have been found, greater neural differences between languages often correspond to greater disparities in skill level^100,101^, consistent with our finding that in multilinguals, English proficiency modulates the extent to which language-specific knowledge is neurally encoded^102–104^ (Fig. 5i).

Studying speech and language in the human brain has traditionally been limited to a small number of languages and typically with native speakers of those languages. Although more work is needed to survey the vast linguistic diversity that exists across the world’s languages, this work shows that broadening the scope of enquiry to a diversity of cultural and experiential differences can give critical insights into fundamental mechanisms of neural coding for communication.

## Methods

Data acquisition followed procedures similar to those conducted in previous work^12,38,106^.

### Participants

Thirty-four patients (17 female) were implanted subdural ECoG grids (Integra or PMT) with 4-mm centre-to-centre electrode spacing and 1.17-mm-diameter contacts (recording tens to hundreds of thousands of neurons^107,108^) as part of their neurosurgical treatment at either University of California, San Francisco (UCSF) or Huashan Hospital. All participants gave informed written consent to participate in the study before experimental testing. Most electrode grids were placed over the lateral surface of a single hemisphere and were centred around the temporal lobe extending to adjacent cortical areas. The precise location of electrode placement was determined by clinical assessment.

For all participants, age, gender, language background information and which hemisphere was recorded from is included in Extended Data Tables 1–4, where each table corresponds to the respective participant group: English monolingual, Spanish and Mandarin monolingual, Spanish–English bilingual and others with diverse language backgrounds. Which participants were included in each set of analyses is listed in Extended Data Table 5. ECoG recordings with the four Mandarin speakers were conducted at Huashan Hospital while patients underwent awake language mapping as part of their surgical brain-tumour treatment. ECoG recordings with all other participants were conducted at UCSF while patients underwent clinical monitoring for seizure activity as part of their surgical treatment for intractable epilepsy. Most participants in this dataset had epileptic seizure foci that were located in deep medial structures (for example, the insula) or far anterior temporal lobe outside of the main regions of interest in this study, such as the STG. All participants included in this study reported normal hearing and spoken language abilities.

### Participant consent

All protocols in the current study were approved by the UCSF Committee on Human Research and by the Huashan Hospital Institutional Review Board of Fudan University. Participants gave informed written consent to take part in the experiments and for their data to be analysed. Informed consent of non-English-speaking participants at UCSF was acquired using a medically certified interpreter platform (Language-Line Solutions), and communication with research staff was facilitated by either in-person or video-call-based interpreters who were fluent in the participant’s native language.

### Language questionnaire

All participants were asked to self-report speech comprehension proficiency, age of acquisition and frequency of use for all languages that they were familiar with. Participants who self-identified as Spanish–English bilingual were asked to complete a comprehensive language questionnaire by means of an online Qualtrics survey^103^.

### Neural data acquisition

ECoG signals were recorded with a multichannel PZ5 amplifier connected to an RZ2 digital signal acquisition system (TuckerDavis Technologies (TDT)) with a sampling rate of 3 kHz. During the stimulus presentation, the audio signal was recorded in the TDT circuit and therefore time-aligned with the ECoG signal. Audio stimulus was also recorded with a microphone, and this signal was also recorded in the TDT circuit to ensure accurate time alignment.

### Data preprocessing

Offline preprocessing of the data included downsampling to 400 Hz, notch-filtering of line noise (at 60 Hz, 120 Hz and 180 Hz for recording at UCSF and 50 Hz, 100 Hz and 150 Hz for recording at Huashan Hospital), extracting the analytic amplitude in the high-gamma frequency range (70–150 Hz, HFA) and excluding extended interictal spiking or otherwise noisy channel activity through manual inspection. The Hilbert transform was used to extract HFA using eight band-pass Gaussian filters with logarithmically increasing centre frequencies (70–150 Hz) and semilogarithmically increasing bandwidths. High-gamma amplitude was calculated as in previous work^12,106^ as the first principal component of the signal in each electrode across all eight high-gamma bands, using principal components analysis (PCA). Last, the HFA was downsampled to 100 Hz and *Z*-scored relative to the mean and s.d. of the neural data in the experimental block. Each sentence HFA was normalized relative to the 0.5 s of silent prestimulus baseline. All subsequent analyses were based on the resulting neural time series.

### Electrode localization

For anatomical localization, electrode locations were extracted from postimplantation computer tomography scans, coregistered to the patients’ structural magnetic resonance imaging and superimposed on three-dimensional reconstructions of the patients’ cortical surfaces using an in-house imaging pipeline validated in previous work^109^. FreeSurfer (https://surfer.nmr.mgh.harvard.edu/) was used to create a three-dimensional model of the individual participant’s pial surfaces, run automatic parcellation to get individual anatomical labels and warp the individual participant surfaces into the cvs_avg35_inMNI152 average template.

### Stimuli and procedure

All participants passively listened to roughly 30 min of speech in a language they knew (either Spanish, English or Mandarin Chinese) and one other language (either Spanish or English). Speech stimuli consisted of a selection of 239 unique Spanish sentences from the DIMEx corpus^30,110^, spoken by a variety of native Mexican-Spanish speakers; 499 unique English sentences from the TIMIT corpus, spoken by a variety of American English speakers^31^; and 58 unique Mandarin Chinese paragraphs from the ASCCD corpus from the Chinese Linguistic Data Consortium (www.chineseldc.org/), spoken by a variety of Mandarin Chinese speakers. Although the total duration of stimuli presentation was roughly equal across languages, statistical tests that were conducted at the sentence level across corpora accounted for the differing number of sentences.

We selected these specific speech corpora, originally designed to test speech recognition systems, to meet the following requirements: (1) comprehensively span the phonemic inventories of the languages in question; (2) provide validated phonemic, phonetic and word-level annotations; and (3) include a diverse and wide range of speakers with varying accents. These corpora were not intended to span specific semantic or syntactic structures in the languages or to capture long-range dependencies that extended across multiple sentences. Therefore we did not ask research questions about these specific speech representations.

The contents of each speech corpus were split across five blocks, where each block was roughly 5–7 minutes in duration. In English and Spanish speech stimuli, four blocks contained unique sentences, and a single block contained ten repetitions of ten sentences. In the Mandarin Chinese speech stimuli, four paragraphs were repeated six times, and repetitions were intermixed with unique paragraphs across blocks. Repeated sentences were used for validation of TRF models (see details below). Sentences were presented with an intertrial interval of 0.4–0.5 s in English and Mandarin and 0.8 s in Spanish. Spanish sentences were on average 4.77 s long (range: 2.5–8.03 s), English sentences were on average 3.05 s long (range: 1.99–3.60 s), and Mandarin Chinese sentences were on average 3.16 s long (range: 1.17–11.76 s). Although speech blocks of different languages could be intermixed in the same recording session, each 5- to 7-min block of speech consisted of only one language.

Speech stimuli were presented at a comfortable ambient loudness (about 70 dB) through free-field speakers placed roughly 80 cm in front of the patients’ head. All speech stimuli were presented in the experiment using custom-written MATLABR2016b scripts (MathWorks, www.mathworks.com). Participants were asked to listen to the stimuli attentively and were free to keep their eyes open or closed during the stimulus presentation. We performed all subsequent data analysis using custom-written MATLABR2024b scripts.

### Electrode selection

Subsequent analyses included all speech-responsive electrodes: electrodes for which at least a contiguous 0.1 s of the neural response (10 samples, 100-Hz sampling rate) time-aligned to the first or last 0.5 s of the spoken sentences was significantly different from that of the prespeech silent baseline. To test for significance, we used a one-way ANOVA *F*-test at each time point, Bonferroni corrected for multiple comparisons using a threshold of *P* < 0.01. We included the neural response aligned to the last 0.5 s of each sentence to ensure that we did not exclude electrodes for which there was a significant response time aligned to the end of sentences but not the beginning.

### Feature TRF analysis

HFA responses to speech corpora were predicted using standard linear TRF models with various sets of speech features^37,111,112^. Many of the speech features used were extracted from pre-existing corrected acoustic, phonetic, phonemic and word-level annotations included with these speech corpora. In the feature TRF (F-TRF) models described below, HFA neural responses recorded from a single electrode were predicted as a linear combination of speech features that occurred at most 0.6 s before the current time point:

$${\rm{H}}{\rm{F}}{\rm{A}}(t)={x}_{0}+\mathop{\sum }\limits_{f=1}^{F}\mathop{\sum }\limits_{l=1}^{L}\beta (l,\,f)X(f,\,t-l)$$

where *x*_0_ corresponds to the model intercept, *F* corresponds to the set of speech features included in the model and *L* corresponds to the number of time lags considered in the model (60 samples reflecting a total lag of 0.6 s). *β*(*l*,* f*) weights are therefore interpreted as the weight or importance of the specific combination of speech feature, *f*, and time-lag, *l*, in predicting the final neural response given the set of speech features and training sentences. Models were trained and tested separately for each electrode using the same cross-validation, ridge regularization and feature normalization protocols as reported in previous work^12,38,106^. To derive the F-TRF model performance, we fit the trained model to a held-out set of roughly ten sentences averaged across ten repeats (see ‘Stimuli and procedure’) and then calculated the correlation-based *R*^2^ fit. In subsequent analysis, we analysed both the magnitude *β* weights for each speech feature and the overall model *R*^2^ derived from the held-out test sentences. In addition to training and testing F-TRF models on sentences in the same language, we also trained F-TRF models on a language and tested the model on sentences from another language to test the generality of our model fits (Extended Data Fig. 5). This analysis followed the same training and testing procedure as the models that were trained and tested on the same language.

### Selection of features for F-TRF

The speech features included in the acoustic–phonetic F-TRF model were selected from those that have been identified in previous studies to strongly drive HFA in the human temporal cortex. We included the sentence onset feature as well as the acoustic edges of envelope (peakRate^38^), both features that drive neural responses in the posterior STG and middle STG, respectively. The consonant manner of articulation and place of articulation features were selected on the basis of ref. ^2^, which included binary values at phoneme onset indicating whether the phoneme was dorsal, coronal, labial, nasal, plosive or fricative. The vowel features were selected on the basis of ref. ^106^, which included the median formant values for F1, F2, F3 and F4 (Hz) for each vowel sound because continuous formant values were shown to drive neural responses more than categorical vowel features (for example, high, front, low, back).

To test the effect of word-level features on the F-TRF neural prediction, we also included word onset, word frequency, absolute word length (as number of phonemes) and within-word phoneme surprisal. All features had been pre-annotated for each speech corpus except for word frequency, phoneme surprisal and syllable onset (in Spanish only). Word boundary and phoneme surprisal are metrics reflective of how the brain tracks sequential speech structure, with the former being a discrete, sparse marker of segmentation and the latter being a continuous measure of how predictable each phoneme is, given all preceding phonemes in the current word. Word-frequency and phoneme-surprisal features were calculated on the basis of the distribution of the language presented regardless of the participant’s language background (Spanish surprisal was used in the model in Spanish speech blocks; a precise description of how these features were generated is below). In the TRF model, we excluded phoneme surprisal at word initial positions so that the word-level features could be dissociated from the phoneme-surprisal feature in time.

### Model weight correlation across conditions

We determined a particular electrode’s feature tuning by analysing the model weights, where each weight was proportional to the change in electrode response given a change in that feature’s value^112^. We estimated the F-TRF *β* weights corresponding to each unique speech feature for each electrode separately, as described in the model-fitting procedure above^2^. To compare electrode encoding of the base F-TRF model across speech conditions (Fig. 1), we first selected all electrodes with *R*^2^ > 0.1 for both speech conditions; this ensured that the *β* weight analysis would be based on TRF models that were able to explain the neural responses sufficiently well. We then extracted the mean of the 0.05-s (five samples) window around the maximum *β* weight averaged across features. This resulted in a single vector for each electrode per speech condition, with one *β* weight per speech feature; to calculate weight correlation, we performed a Pearson correlation across vectors of the same electrode from different speech conditions. This allowed us to determine the cross-language correlation between the relative *β* weights. To determine a distribution at chance level across electrodes, we performed permutation testing by shuffling the *β* weights for a single speech condition and repeating the Pearson correlation ten times per electrode.

### Construction of word-frequency, word-length and within-word phoneme-surprisal features

Word-frequency values were adopted from the SUBTLEX American English^113^ and SUBTLEX Spanish^51^ frequency estimates, which included a total of 74,287 and 94,341 unique words respectively. We included frequency estimates for all words in our corpora that existed in the English and Spanish SUBTLEX word sets. All word-frequency measures were log-scaled (base 10). Word length was determined as the number of phonemes contained within the word onset and offset from the corpus annotations. We used the same word frequency and word lists documented in the SUBTLEX American English and Spanish corpus to construct within-word phoneme surprisal, where each phoneme was associated with a surprisal value. We constructed within-word phoneme surprisal as

$$-{\rm{l}}{\rm{o}}{{\rm{g}}}_{2}\,\left[P({x}_{n}|{x}_{1}\ldots {x}_{n-1})=\frac{{\rm{f}}{\rm{r}}{\rm{e}}{\rm{q}}({x}_{1}\ldots {x}_{n})}{{\rm{f}}{\rm{r}}{\rm{e}}{\rm{q}}({x}_{1}\ldots {x}_{n-1})}\right]$$

where ${x}_{1}\ldots {x}_{n-1}$ is all preceding phonemes in the current word and *x*_*n*_ is the current phoneme. We did not include surprisal values for the first phoneme of a word, only phonemes at the second position in the word onwards. $\mathrm{freq}({x}_{1}\ldots {x}_{n})$ is the count of all unique words with ${x}_{1}\ldots {x}_{n}$ as the prefix multiplied by the frequency of each of those words as estimated by the SUBTLEX English and Spanish corpus. We also calculated within-word phoneme entropy (expected value of surprisal at each phoneme) as well as biphone and triphone surprisal and entropy but ultimately did not include these features in the final F-TRF model, as for most electrodes, these features did not explain added unique variance.

### Annotation of DIMEx syllable onset

We generated the syllable-onset feature for the DIMEx speech corpus through a combination of automatic syllable-onset detection and manual validation. We did not annotate instances of resyllabified word onsets, such as instances where the consonants at the end (coda) of a syllable were pronounced as part of the onset of the following syllable. For example, the phrase *los otros* (los.'otros) would be annotated to reflect the canonical form (los.'otros), although it may be pronounced (lo.'so.tros).

### Unique variance calculation

Because several of the speech features we used in the F-TRF models were highly correlated, we determined the unique contribution of each feature to driving neural responses through the calculation of unique variance. Unique variance $(\Delta {R}^{2})$ is defined as the difference between the portion of variance explained by the model with all relevant features and the model with the feature of interest (*f*) removed. Broadly, this is

$$\Delta {R}^{2}(f)=[\mathrm{full}\,\mathrm{model}]{R}^{2}-[\mathrm{model}\,\mathrm{with}\,f\,\mathrm{removed}]{R}^{2}$$

The acoustic–phonetic F-TRF model included sentence onset, peakRate, consonant features (dorsal, coronal, labial, nasal, plosive, fricative) and vowel formants (F1, F2, F3, F4). The full F-TRF model included sentence onset, peakRate, consonant features (dorsal, coronal, labial, nasal, plosive, fricative), vowel formants (F1, F2, F3, F4), word onset, word frequency, word length and phoneme-surprisal.

Unique variance for acoustic–phonetic features (Fig. 2) was calculated as follows:

$$\Delta {R}^{2}(\mathrm{sentence}\,\mathrm{onset})=[\mathrm{full}\,\mathrm{model}]{R}^{2}-[\mathrm{full}\,\mathrm{model}-\mathrm{sentence}\,\mathrm{onset}]{R}^{2}$$

$$\Delta {R}^{2}(\mathrm{peakRate})=[\mathrm{full}\,\mathrm{model}]{R}^{2}-[\mathrm{full}\,\mathrm{model}-\mathrm{peakRate}]{R}^{2}$$

$$\Delta {R}^{2}(\mathrm{consonant}\,\mathrm{features})=[\mathrm{full}\,\mathrm{model}]{R}^{2}-[\mathrm{full}\,\mathrm{model}-\mathrm{consonant}\,\mathrm{features}]{R}^{2}$$

$$\Delta {R}^{2}(\mathrm{vowel}\,\mathrm{formants})=[\mathrm{full}\,\mathrm{model}]{R}^{2}-[\mathrm{full}\,\mathrm{model}-\mathrm{vowel}\,\mathrm{formants}]{R}^{2}$$

Unique variance for word-level and phoneme-surprisal features (Fig. 2) was calculated as follows:

$$\Delta {R}^{2}(\mathrm{word}\,\mathrm{onset})=[\mathrm{acoustic}\,\mathrm{phonetic}\,\mathrm{model}+\mathrm{word}\,\mathrm{onset}]{R}^{2}-[\mathrm{acoustic}\,\mathrm{phonetic}\,\mathrm{model}]{R}^{2}$$

$$\Delta {R}^{2}(\mathrm{word}\,\mathrm{frequency})=[\mathrm{acoustic}\,\mathrm{phonetic}\,\mathrm{model}+\mathrm{word}\,\mathrm{onset}+\mathrm{word}\,\mathrm{frequency}]{R}^{2}-[\mathrm{acoustic}\,\mathrm{phonetic}\,\mathrm{model}+\mathrm{word}\,\mathrm{onset}]{R}^{2}$$

$$\Delta {R}^{2}(\mathrm{word}\,\mathrm{length})=[\mathrm{acoustic}\,\mathrm{phonetic}\,\mathrm{model}+\mathrm{word}\,\mathrm{onset}+\mathrm{word}\,\mathrm{length}]{R}^{2}-[\mathrm{acoustic}\,\mathrm{phonetic}\,\mathrm{model}+\mathrm{word}\,\mathrm{onset}]{R}^{2}$$

$$\Delta {R}^{2}(\mathrm{phoneme}\,\mathrm{surprisal})=[\mathrm{acoustic}\,\mathrm{phonetic}\,\mathrm{model}+\mathrm{phoneme}\,\mathrm{surprisal}]{R}^{2}-[\mathrm{acoustic}\,\mathrm{phonetic}\,\mathrm{model}]{R}^{2}$$

Combined unique variance for all word-level and phoneme-surprisal features was calculated as:

$$\begin{array}{c}\Delta {R}^{2}(\mathrm{word}\,\mathrm{features})\\ \,=\,[\mathrm{full}\,\mathrm{model}]{R}^{2}-[\mathrm{acoustic}\,\mathrm{phonetic}\,\mathrm{model}]{R}^{2}\end{array}$$

To determine the significance of unique variance values for a single feature or feature family, we computed a distribution of 300 permuted unique variance values by applying random circular temporal shifts to the feature or feature family of interest. To acquire the *P* value associated with the unique variance value, we summed the number of permuted unique values greater than the true unique value and divided by the total number of permutations we computed.

### Statistical testing

LME models as computed by the fitlme function in MATLABR2024b included random effects for participant and hemisphere as well as the interaction between participant and electrode to ensure that the effects we found were significant across participants and hemispheres. LME models were primarily used to determine whether the unique variance of a particular feature *f* was significantly different between native and foreign speech conditions. LME formulas took the following form: Δ*R*^2^(*f*) ~ speech condition + speaker language + (1|hemisphere) + (1|participant) + (1|electrode:participant).

The same LME formula (above) was used to test significance in instances in which TRF feature unique variance could be compared across speech conditions (for example, Figs. 2b,c and 5d).

For Pearson and Spearman correlation, two-tailed tests were performed unless otherwise noted.

### Acoustic word-boundary decoding

Acoustic word-boundary decoding consisted of a logistic regression model trained to classify whether the mel-spectrogram from 0.2 s around a boundary event was a within-word syllable boundary or a word boundary. All single-syllable words and words at sentence onset were removed from this analysis. To match the instances of word and syllable boundaries in the Spanish and English corpora, we randomly sampled 1,900 instances of word and within-word syllable boundaries from each corpus. In Spanish, we used 1,273 within-word syllable onsets and 627 word onsets. In English, we used 1,153 within-word syllable onsets and 747 word onsets.

For each boundary event in both corpora, the 0.2-s 80-band mel-spectrogram (sampled at 100 Hz) was vectorized. PCA was then performed on the vectorized mel-spectrogram to reduce the total dimensionality. The components with the highest variance that cumulatively explained more than 90% of the total variance were ultimately used as input to the regression, which totalled about 60–80 PCs depending on the language. Logistic regression was run using standard MATLABR2024b regression functions (fitclinear) with Lasso (L1) regularization and 20-fold cross-validation. The AUC was extracted for each held-out test fold, where chance AUC would equal 0.5.

In addition to training and testing acoustic word-boundary decoding in the same language, we also trained the word-boundary decoders in one language and tested on boundary events from the other language to test the extent to which acoustic cues to word boundaries overlapped between the languages. This analysis followed the same training and testing procedure as for the decoders that were trained and tested on the same language. To assess the significance of the test-set AUC values, we performed permutation testing in which word and within-word syllable boundary labels were randomly shuffled and the same logistic regression procedure was applied.

### Neural word-boundary effects

To determine whether speech-responsive electrodes showed a significant difference between boundary events (within-word syllable boundaries and word boundaries), we assessed the duration of contiguous time points for which the neural response (100 Hz) time aligned 0.2 s before and 0.4 s after word-boundary events significantly differed from responses aligned to within-word syllable boundaries. To test for significance, we used a one-way ANOVA *F*-test at each time point and Bonferroni corrected for multiple comparisons using a threshold of *P* < 0.01. We computed this for each electrode and each language separately such that every electrode was associated with the duration of significant differences value for each language.

### Neural word-boundary decoding

Neural word-boundary decoding consisted of a logistic regression model trained to classify whether the contiguous window of neural activity 0.2 s before and 0.4 s after a boundary corresponded to a within-word syllable boundary or a word boundary. To compare against the acoustic word-boundary decoding, all single-syllable words and words at sentence onset were removed from this analysis. The size of the window used in neural decoding was larger than that of the acoustic decoding (0.2 s) because the neural response dynamics are slower in latency than the acoustic dynamics.

To perform neural word-boundary decoding, neural responses (sampled at 100 Hz) from all speech-responsive electrodes from a subset of participants were concatenated into a matrix of the form (electrode × time × event), which was then vectorized across the electrode and time dimensions. The subset of participants who had the maximum overlap in trials were used for neural word-boundary decoding. We performed PCA on the vectorized spatiotemporal neural data to reduce the total dimensionality. The components with the highest variance that cumulatively explained more than 90% of the total variance were ultimately used as input to the regression: about 800–1,000 PCs, depending on the participant group. Extended Data Table 6 summarizes the number of {unique participants, electrodes, PCs} used to train each decoder.

Logistic regression was run using standard MATLABR2024b regression functions (fitclinear) with Lasso (L1) regularization and 20-fold cross-validation. The AUC was extracted for each held-out test fold. Beta linear coefficient estimates were extracted from the trained ClassificationLinear model, and the weights of single electrodes were calculated by thresholding the percentile weight values and converting from PC space back to the original (electrode × time) space.

To characterize the temporal dynamics of decoding performance (Extended Data Fig. 9), we performed sliding-window neural word-boundary decoding. To do this, we used the same set of speech-responsive electrodes as for the fixed 0.6-s window decoding, but we used a sliding window of 0.02 s within this 0.6 s of neural response to perform the decoding. The same trials and electrodes were used for decoding across all 0.02-s windows in the same participant group and language.

We also performed single-participant neural word-boundary decoding (Extended Data Fig. 9) using all speech-responsive electrodes per participant. Single-participant word-boundary decoding was calculated similarly to the participant group decoding (with the same time window, with PCA) but included electrode responses from a single participant only. The number of electrodes included in the decoding varied by participant (electrodes: median = 47, range = {21, 79}). For statistical analysis comparing decoding performance across speech conditions in single participants (Extended Data Fig. 9c), a paired Wilcoxon signed-rank test was used.

For the single participants who spoke English and at least one other language (Fig. 5), we followed the same procedure as above for calculating English word-boundary decoding AUC. To assess the significance of the effect of English proficiency on neural word-boundary classification performance (in English), we used a linear model:

$$\mathrm{AUC} \sim \mathrm{proficiency}+(1|\mathrm{number}\,\mathrm{of}\,\mathrm{electrodes})$$

### Construction of AAI

The acoustic ambiguity index (AAI) is based on the acoustic word-boundary decoder that takes in the spectrogram values using 0.2 s around each boundary event and outputs whether the trial corresponds to a word boundary or a within-word syllable boundary. As above, single-syllable words and words at sentence onset were removed from this analysis. For each single trial, the decoder outputs both a predicted label (0, 1) and a posterior probability score (0–1). The scores of the held-out test trials are extracted as one of the outputs of the MATLABR2024b ClassificationLinear model predict function. The per-trial value abs(correct label – posterior probability score) is then calculated to produce a continuous measure of how accurate the classifier is on single trials, where larger values indicate that the classifier is probably predicting incorrectly. The corresponding neural word-boundary decoding is produced after (1) calculating the difference between the probability scores for single trials from neural decoding and correct label and (2) comparing this distribution between known and foreign groups for each bin.

### Reporting summary

Further information on research design is available in the Nature Portfolio Reporting Summary linked to this article.

## Online content

Any methods, additional references, Nature Portfolio reporting summaries, source data, extended data, supplementary information, acknowledgements, peer review information; details of author contributions and competing interests; and statements of data and code availability are available at 10.1038/s41586-025-09748-8.

## Supplementary information

Reporting Summary

## Figures and Tables

**Extended Data Table 1:**
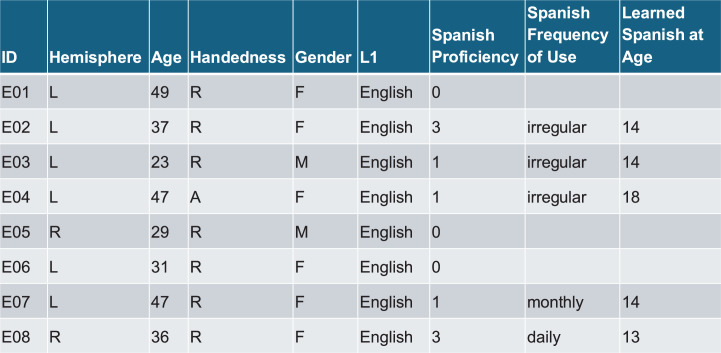
Spanish proficiency, frequency of use, and age of acquisition for self-reported English monolingual participants

Self-rated proficiency profiles of Spanish for English monolingual participants. (L1: First learned language). Proficiency scale as: 1- single words, 2-basic conversation, 3-fluent, 4-very good, 5- native. Frequency of use scale as using this language: 1-daily, 2-weekly, 3-monthly, 4-irregular, 5-never.

**Extended Data Table 2:**
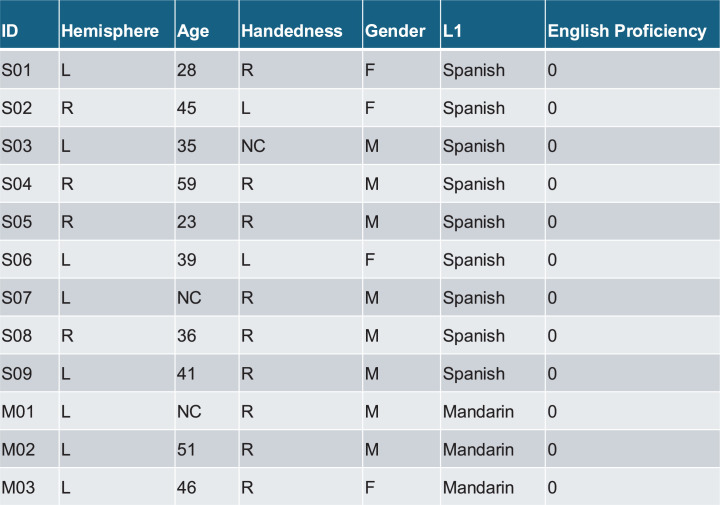
English proficiency, frequency of use, and age of acquisition for self-reported Spanish and Mandarin monolingual participants

Self-rated proficiency profiles of English for Spanish and Mandarin monolingual participants. (L1: First learned language, NC: Not collected). Proficiency scale as: 1- single words, 2-basic conversation, 3-fluent, 4-very good, 5- native. Frequency of use scale as using this language: 1-daily, 2-weekly, 3-monthly, 4-irregular, 5-never.

**Extended Data Table 3:**
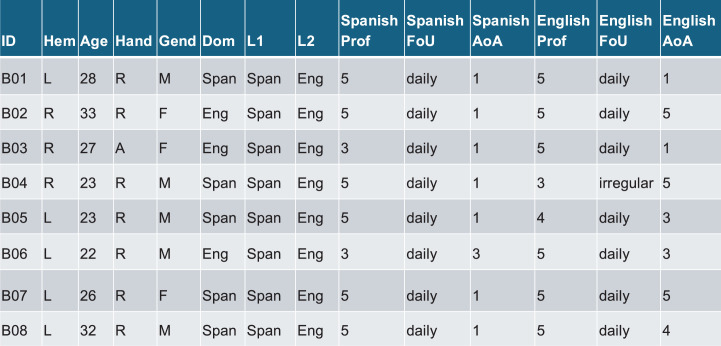
Spanish and English proficiency, frequency of use, and age of acquisition for self-reported Spanish-English bilinguals

Self-rated Spanish and English proficiency profiles of Spanish-English multilingual participants (Hem: hemisphere, Hand: Handedness, Gend: Gender, Dom: Dominant Language, FoU: Frequency of Use, AoA: Age of Acquisition, Span: Spanish, Eng: English, Cat: Catalan, NC: Not collected). Proficiency scale as: 1- single words, 2-basic conversation, 3-fluent, 4-very good, 5- native. Frequency of use scale as using this language: 1-daily, 2-weekly, 3-monthly, 4-irregular, 5-never.

**Extended Data Table 4:**
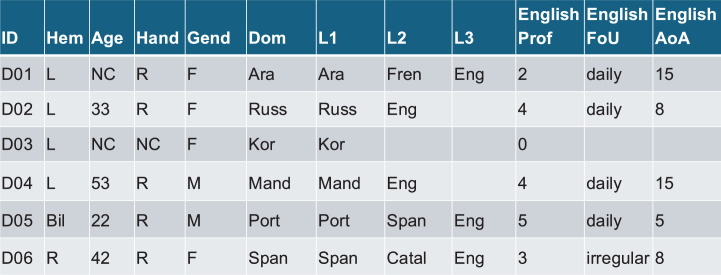
English proficiency, frequency of use, and age of acquisition for participants with diverse language backgrounds

Self-rated English proficiency profiles of diverse multilingual participants (Hem: hemisphere, Hand: Handedness, Gend: Gender, Dom: Dominant Language, FoU: Frequency of Use, AoA: Age of Acquisition, Span: Spanish, Eng: English, Cat: Catalan, Port: Portuguese, Mand: Mandarin, Kor: Korean, Ara: Arabic, Russ: Russian, NC: Not collected). Proficiency scale as: 1- single words, 2-basic conversation, 3-fluent, 4-very good, 5- native. Frequency of use scale as using this language: 1-daily,2-weekly, 3-monthly, 4-irregular, 5-never.

**Extended Data Table 5:**
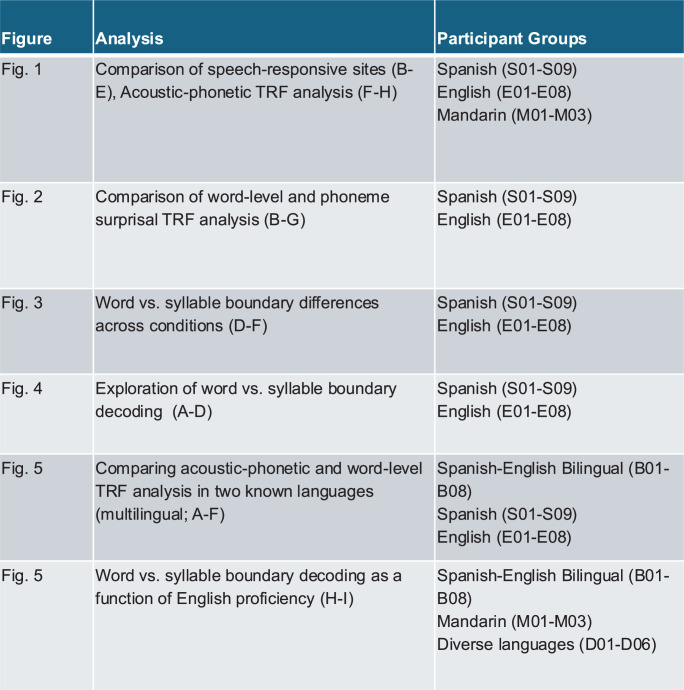
Summary of participant groups in each analysis

Participants cohorts included in each analysis within each of the main figures.

**Extended Data Table 6:**
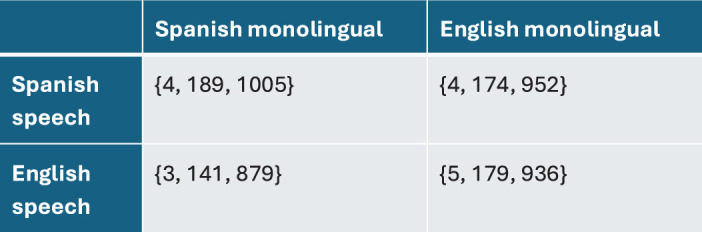
Summary of participants, electrodes, PCs used in each decoder

The number of participants, PCs, and trials included in each neural word boundary classification run across speaker types (Spanish, English monolinguals) and speech condition (English, Spanish speech).

## Data Availability

Data to replicate all figures are available at Zenodo (10.5281/zenodo.17247450)^114^ and are available on request to the corresponding author. Because participants at the outset of this study had not consented for public data release, these data are only available upon reasonable request to respect patient privacy and consent.

## Code availability

All MATLABR2024b codes used to generate the main and Extended Data figures are available at https://github.com/ChangLabUcsf/BhayaGrossman2025.
