# Supplementary material for: Shared and language-specific phonological processing in the human temporal lobe
Source: Nature. 2025 Nov 19;649(8095):140–51. doi: 10.1038/s41586-025-09748-8 (PMC12727522; doi:10.1038/s41586-025-09748-8)
Supplement: Supplementary file 1 — Reporting Summary [file 41586_2025_9748_MOESM1_ESM.pdf]

Corresponding author(s): Edward Chang
Last updated by author(s): Sep 30, 2025

Reporting Summary

Nature Portfolio wishes to improve the reproducibility of the work that we publish. This form provides structure for consistency and transparency in reporting. For further information on Nature Portfolio policies, see our Editorial Policies and the Editorial Policy Checklist.

Statistics

For all statistical analyses, confirm that the following items are present in the figure legend, table legend, main text, or Methods section.

n/a	Confirmed	
☐	☒	The exact sample size (*n*) for each experimental group/condition, given as a discrete number and unit of measurement
☐	☒	A statement on whether measurements were taken from distinct samples or whether the same sample was measured repeatedly
☐	☒	The statistical test(s) used AND whether they are one- or two-sided<br>*Only common tests should be described solely by name; describe more complex techniques in the Methods section.*
☐	☒	A description of all covariates tested
☐	☒	A description of any assumptions or corrections, such as tests of normality and adjustment for multiple comparisons
☐	☒	A full description of the statistical parameters including central tendency (e.g. means) or other basic estimates (e.g. regression coefficient) AND variation (e.g. standard deviation) or associated estimates of uncertainty (e.g. confidence intervals)
☐	☒	For null hypothesis testing, the test statistic (e.g. *F*, *t*, *r*) with confidence intervals, effect sizes, degrees of freedom and *P* value noted<br>*Give P values as exact values whenever suitable.*
☒	☐	For Bayesian analysis, information on the choice of priors and Markov chain Monte Carlo settings
☒	☐	For hierarchical and complex designs, identification of the appropriate level for tests and full reporting of outcomes
☐	☒	Estimates of effect sizes (e.g. Cohen's *d*, Pearson's *r*), indicating how they were calculated

Our web collection on statistics for biologists contains articles on many of the points above.

Software and code

Policy information about availability of computer code

Data collection	Data was recorded using Tucker-Davis Technologies (TDT) proprietary system and software. All speech stimuli were presented in the experiment using custom-written MATLAB2016b scripts [MathWorks, www.mathworks.com].
Data analysis	These data were preprocessed using custom MATLAB2014a and python scripts, some of which can be found at: https://github.com/ChangLabUcsf/img_pipe. Electrode localization and reconstruction was performed using FreeSurfer 7.2.0 and the package from [https://github.com/ChangLabUcsf/img_pipe]. All subsequent data analysis and visualization were performed in MATLAB2024b using built-in scientific toolboxes, open-source community scripts, and custom-built scripts. The full analysis code is available at: https://github.com/ChangLabUcsf/BhayaGrossman2025, which contains documentation for all scripts and workflows.

For manuscripts utilizing custom algorithms or software that are central to the research but not yet described in published literature, software must be made available to editors and reviewers. We strongly encourage code deposition in a community repository (e.g. GitHub). See the Nature Portfolio guidelines for submitting code & software for further information.

Data

Policy information about availability of data

All manuscripts must include a data availability statement. This statement should provide the following information, where applicable:

- Accession codes, unique identifiers, or web links for publicly available datasets
- A description of any restrictions on data availability
- For clinical datasets or third party data, please ensure that the statement adheres to our policy

Data to replicate all figures is posted to Zenodo (10.5281/zenodo.17247450) and is available on request to the corresponding author (Edward.Chang@ucsf.edu). Because participants at the outset of this study were not consented for public data release, these data are only available upon reasonable request to respect patient privacy and consent.

Research involving human participants, their data, or biological material

Policy information about studies with human participants or human data. See also policy information about sex, gender (identity/presentation), and sexual orientation and race, ethnicity and racism.

Reporting on sex and gender	Our study includes data collected from approximately equal numbers of male and female participants (of the 31 main participants, 15 participants self-reported as female). Sex was determined based on self-report. We did not analyze the data separately for males and females because we did not predict differences in our results based on sex.
Reporting on race, ethnicity, or other socially relevant groupings	Race and ethnicity were not assessed in this study. Therefore no data on these variables are reported.
Population characteristics	This study included only participants diagnosed with intractable epilepsy. Data collection was performed with patients who had undergone an implantation procedure and were being monitored in the hospital as part of their clinical care. While adults with epilepsy can exhibit language deficits, we only collected data from patients who reported normal language function, both in production and in comprehension.
Recruitment	Participants were recruited from the Neurosurgical Department from the University of California, San Francisco and Fudan University, Shanghai. All patients who met the inclusion criteria (e.g. speakers of a non-English language with relevant neuro-anatomical coverage) and consented to participate in research were included in the study. To the best of our knowledge, we did not introduce biases into our recruitment process.
Ethics oversight	All protocols in the current study were approved by the University of California, San Francisco Committee on Human Research and by the Huashan Hospital Institutional Review Board of Fudan University. Participants gave informed written consent to take part in the experiments and for their data to be analyzed. Informed consent of non-English speaking participants at UCSF was acquired via a medically certified interpreter platform (Language-Line Solutions) and communication with research staff was facilitated by either in-person or video-call based interpreters who were fluent in the participant's native language.

Note that full information on the approval of the study protocol must also be provided in the manuscript.

Field-specific reporting

Please select the one below that is the best fit for your research. If you are not sure, read the appropriate sections before making your selection.

☒ Life sciences ☐ Behavioural & social sciences ☐ Ecological, evolutionary & environmental sciences

For a reference copy of the document with all sections, see nature.com/documents/nr-reporting-summary-flat.pdf

Life sciences study design

All studies must disclose on these points even when the disclosure is negative.

Sample size	The sample size (n=34 participants) was determined by the number of eligible patients who consented to participate in the study over a period of 10 years. This approach was chosen since this study was conducted in a clinical setting, where recruitment was reliant on patient consent and participation.<br><br>To inform the study design, we reviewed prior literature in which electrocorticography (ECoG) was used, which reported sample sizes ranging from 5 to 25 in similar studies (e.g. Sjerps, Fox, Johnson, & Chang (2019) Nature Communications; Hamilton, Edwards, & Chang (2018) Current Biology; Cogan et al (2014) Nature). Based on these reports, we estimated that a sample size of approximately 10 participants per group would be sufficient to detect statistically significant differences.
Data exclusions	Neurophysiological data was excluded from analysis if pathological neural activity (namely, interictal epileptic discharges) was identified either during data collection or prior to analysis during visual inspection.
Replication	To assess the reproducibility of our findings, we conducted independent experiments with different participant cohorts (English speakers,

Replication	Spanish speakers, English-Spanish bilinguals, Mandarin speakers). The key findings of the study, namely that the neural encoding of acoustic phonetic features is preserved across native and foreign languages but for word-level features is enhanced, were replicated across all three independent cohorts.
Randomization	Randomization was not relevant in the present study, as participants were included based on language background. Covariates such as the hemisphere from which neural data was recorded, were controlled statistically in linear mixed effect models as random effects.
Blinding	Investigators were not blinded to the group allocation since data collection involved directly speaking with the participants and therefore being privy to their language background (the primary feature of interest).

Reporting for specific materials, systems and methods

We require information from authors about some types of materials, experimental systems and methods used in many studies. Here, indicate whether each material, system or method listed is relevant to your study. If you are not sure if a list item applies to your research, read the appropriate section before selecting a response.

Materials & experimental systems

n/a	Involved in the study
☒	☐ Antibodies
☒	☐ Eukaryotic cell lines
☒	☐ Palaeontology and archaeology
☒	☐ Animals and other organisms
☒	☐ Clinical data
☒	☐ Dual use research of concern
☒	☐ Plants

Methods

n/a	Involved in the study
☒	☐ ChIP-seq
☒	☐ Flow cytometry
☒	☐ MRI-based neuroimaging

Plants

Seed stocks	N/A
Novel plant genotypes	N/A
Authentication	N/A
